# Naked cuticle homolog 2 controls the differentiation of osteoblasts and osteoclasts and ameliorates bone loss in ovariectomized mice

**DOI:** 10.1016/j.gendis.2024.101209

**Published:** 2024-01-12

**Authors:** Liying Shan, Xiaoxia Liao, Xiaoli Yang, Endong Zhu, Hairui Yuan, Jie Zhou, Xiaoxia Li, Baoli Wang

**Affiliations:** a NHC Key Lab of Hormones and Development and Tianjin Key Lab of Metabolic Diseases, Tianjin Medical University Chu Hsien-I Memorial Hospital & Institute of Endocrinology, Tianjin 300134, China; b College of Basic Medical Sciences, Tianjin Medical University, Tianjin 300070, China

**Keywords:** Mechanistic target of rapamycin complex 1, Naked cuticle homolog 2, Osteoblast, Osteoclast, Receptor activator of nuclear factor κB ligand, Wnt/β-catenin

## Abstract

Naked cuticle homolog 2 (NKD2) has been recognized as an antagonist of Wnt/β-catenin signaling and a tumor suppressor. The role of NKD2 in osteoblast and osteoclast differentiation and the mechanism are not fully understood. In this study, we identified the up-regulation of NKD2 during osteoblast and adipocyte differentiation. Functional experiments revealed that NKD2 stimulated osteoblast differentiation and suppressed adipocyte formation. Furthermore, NKD2 down-regulated the expression of receptor activator of nuclear factor-κB ligand in bone marrow mesenchymal stem cells and inhibited osteoclast formation from osteoclast precursor cells. Mechanistic investigations revealed that the regulation of osteoblast and adipocyte differentiation by NKD2 involved Wnt/β-catenin and tuberous sclerosis complex subunit 1 (TSC1)/mechanistic target of rapamycin complex 1 (mTORC1) signaling pathways. Unlike in undifferentiated mesenchymal cells where NKD2 promoted Dishevelled-1 degradation, in the cells differentiating toward osteoblasts or adipocytes NKD2 down-regulated secreted frizzled related protein 1/4 expression and failed to destabilize Dishevelled-1, thereby activating Wnt/β-catenin signaling. Moreover, NKD2 bound to TSC1 and inhibited mTORC1 signaling. Further investigation uncovered an interplay between TSC1/mTORC1 and Wnt/β-catenin signaling pathways. Finally, transplantation of NKD2-overexpressing bone marrow mesenchymal stem cells into the marrow of mice increased osteoblasts, reduced osteoclasts and marrow fat, and partially prevented bone loss in ovariectomized mice. This study provides evidence that NKD2 in mesenchymal stem/progenitor cells reciprocally regulates the differentiation of osteoblasts and adipocytes by modulating Wnt/β-catenin and mTORC1 pathways and inhibits osteoclast formation by down-regulating receptor activator of nuclear factor-κB ligand. It suggests that NKD2 up-regulation may ameliorate postmenopausal bone loss.

## Introduction

Osteoporosis is a metabolic bone disease that occurs due to relatively excessive osteoclastic bone resorption not compensated by osteoblastic bone formation. It is of interest to acquire a better understanding of the processes that fine-tune the differentiation, survival, and activity of bone cells. Osteoblasts are derived from bone marrow mesenchymal stem cells (BMSCs), which also differentiate toward adipocytes, myoblasts, and chondrocytes under appropriate conditions.^1^, ^2^, ^3^ There exists a competing relationship among these differentiation fates, especially between osteoblasts and adipocytes.^4^, ^5^, ^6^ In contrast, osteoclasts originate from the mononuclear lineage of hematopoietic cells.^7^^,^^8^

Among the variety of regulators that positively regulate osteoblast differentiation, the best characterized include a few transcription factors such as runt-related transcription factor-2 and osterix and several signaling pathways such as Wnt/β-catenin and hedgehog pathways.^9^, ^10^, ^11^, ^12^, ^13^ In contrast, the most important transcription factors that drive adipocyte differentiation are peroxisome proliferator-activated receptor γ and CCAAT/enhancer binding proteins.^14^, ^15^, ^16^ For the differentiation of osteoclasts, it requires at least two cytokines, *i.e.*, colony stimulating factor 1 (CSF1) and receptor activator of nuclear factor-κB ligand (RANKL), both of which are produced by mesenchymal stromal cells, osteoblasts, and osteocytes in the bone microenvironment.^17^

Naked cuticle (NKD), encoded by the Drosophila segment polarity gene, is an EF hand protein that acts as an antagonist of the Wnt pathway by directly interacting with the Wnt signalosome component Dishevelled (DVL).^18^^,^^19^ In vertebrates, there are two NKD homologs, *i.e.*, NKD1 and NKD2, both of which can antagonize canonical and non-canonical Wnt signaling pathways.^20^

NKD2 can suppress tumor growth and/or metastasis in osteosarcoma,^21^ esophageal cancer,^22^ and hepatocellular carcinoma,^23^ at least partly attributed to its inhibitory impact on Wnt/β-catenin signaling. It has recently been reported that NKD2 stimulates the differentiation of dental follicle stem/progenitor cells toward osteoblasts through activating Wnt/β-catenin signaling.^24^ It is unknown whether NKD2 in BMSCs has a role in the differentiation of osteoblasts, adipocytes, and osteoclasts.

This study focused on the contributing role of NKD2 in the differentiation of osteoblasts, adipocytes, and osteoclasts, as well as in bone homeostasis. Furthermore, we elucidated the mechanism by which NKD2 works.

## Materials and methods

### Osteoblast and adipocyte differentiation

ST2 and C3H10T1/2 cells were grown in α-MEM supplemented with 10% fetal bovine serum. Primary BMSCs were collected from the long bones of C57BL/6J mice aged 6–8 weeks following a previously published procedure.^25^ The cells at passages 3–5 were cultured in osteogenic medium or adipogenic medium to allow differentiation. Briefly, for osteoblast differentiation, the cells at 80% confluence were cultured for 14–21 days in an osteogenic medium containing an osteogenic cocktail of 50 μg/mL ascorbic acid and 5 mmol/L β-glycerophosphate. For adipogenic differentiation, the cells at 2 days post–confluence were cultured for 3 days in α-MEM containing an adipogenic cocktail of 5 μg/mL insulin, 0.25 mM methylisobutylxanthine, 0.5 μM dexamethasone, and 50 μM indomethacin. The medium was then refreshed, and the cells were further maintained for 2–3 days in α-MEM containing 5 μg/mL insulin.

### Constructs, siRNAs, and transfections

The CDS region of the NKD2 gene was obtained using PCR and incorporated into pcDNA3.1(+) vector at BamHI/EcoRI restriction sites using ClonExpress II Cloning Kit (Vazyme, Nanjing, China). Transfection of the resulting expression construct or vector was performed using JetPRIME transfection reagent (Polyplus, Illkirch, France). Four hours after transfection, the medium was refreshed. Control siRNA and siRNAs targeting NKD2, β-catenin, and tuberous sclerosis complex subunit 1 (TSC1) were obtained from GenePharma (Shanghai, China). Transfection of 20 nmoL/l siRNA was performed using Lipofectamine RNAiMAX (Invitrogen, Carlsbad, CA, USA). Twenty hours following transfection, the medium was refreshed. The sequences of the siRNAs are shown in Table S1.

### Cell growth rate measurement

ST2 cells in a 96-well plate were transfected with NKD2 expression construct or vector for 4 h using JetPRIME transfection reagent. Forty-eight hours after transfection, 10 μL CCK-8 solution (Vazyme, Nanjing, China) was added to the culture. After an additional 3 h of incubation, the cell growth rate was determined by measuring the optical density at 450 nm.

### Lentiviral packaging and infection

The CDS fragment of the NKD2 gene was incorporated into the lentiviral vector pCDH-CMV-MCS-EF1-copGFP (System Biosciences, Mountain View, USA). The packaging of NKD2 or control vector lentivirus was performed by transfecting the plasmids into 293T cells using a lentiviral packaging kit (Genomeditech, Shanghai, China). The supernatant containing the virus was collected and purified, and the viral titer was measured. Primary BMSCs were infected with the viruses at a multiplicity of infection of 20 and induced to allow differentiation.

### Reverse transcription and quantitative PCR (RT-qPCR)

Cellular RNA was extracted using a total RNA extraction and purification kit (Omega, USA), and mouse tissue RNA was extracted using RNAiso Plus (Takara, Dalian, China). First strand cDNA synthesis was carried out in a 20 μL reaction using a kit by TransGen Biotech (Beijing, China) with 0.5 μg of RNA as the template. Quantitative fluorescent PCR was performed using Sybr Green master mix (ABclonal, Wuhan, China). The PCR primers are listed in Table S2. The relative level of each gene was calculated using the comparative Ct formula. β-actin was used as a reference gene.

### Coimmunoprecipitation

Transfection of ST2 cells with the NKD2 expression construct or vector was performed using JetPRIME. Four hours after transfection, the medium was refreshed, and 48 h after transfection, the total protein was extracted with RIPA lysis buffer. The protein extract was incubated with anti-TSC1 (ABclonal, Cat. N: A0720) or IgG at 4 °C overnight, and then protein A/G-coated magnetic beads were added to capture the immunocomplexes. Following 2 h of incubation, the mixture was washed with a phosphate-buffered saline solution containing 0.5 % Triton® X100 and then boiled with SDS‒PAGE loading buffer. The supernatant was separated from the beads and run on SDS‒PAGE, followed by western blotting to detect NKD2 and TSC1 proteins.

### Protein degradation assay

ST2 cells were transfected with NKD2 expression construct or vector. Twenty-four hours after transfection, the cells were cultured in common medium for 24 h or osteogenic medium for 48 h and then treated with 100 μg/mL cycloheximide for 1, 2, 4, and 6 h. The protein levels of Dishevelled 1 (DVL1) and TSC1 were determined by western blotting.

### Western blotting

Cellular protein extraction was performed using RIPA buffer. Twenty micrograms of protein were separated by 10%–15% SDS‒PAGE and then transferred to nitrocellulose membranes. The membranes were blocked with skim milk and then incubated sequentially with primary antibodies at 4 °C overnight and with horseradish peroxidase-conjugated IgG at room temperature for 1.5 h. The protein bands were developed using a chemiluminescence reagent (ABclonal, Wuhan, China). The primary antibodies used are listed in Table S3.

### Staining of osteoblasts

Differentiated osteoblasts following 14 days of osteogenic induction were fixed with 4% paraformaldehyde for 10 min, washed with phosphate-buffered saline solution, and then stained for alkaline phosphatase (ALP) with NBT/BCIP staining solution (Beyotime, Shanghai, China) for 15 min. For mineralized nodule detection, the cells following 21 days of osteogenic induction were fixed, washed with phosphate-buffered saline solution, and then stained with 0.1% alizarin red S solution (pH 4.2) for 1 h.

### Staining of adipocytes

Differentiated adipocytes following 5–6 days of adipogenic induction were fixed with 4% paraformaldehyde for 10 min, washed with 60% isopropanol, and then stained with 0.24% (*w/v*) oil red O solution for 5 min. The stained adipocytes were observed under a light microscope. The intensity of staining was quantified by extracting the stain from the cells with 100% isopropanol and measuring the optical density at 520 nm.

### Immunofluorescence staining

Transfected cells were fixed with 4% paraformaldehyde for 10 min, washed with phosphate-buffered saline solution, and then permeabilized with 1% Triton® X-100 for 15 min. After blocking with 1% bovine serum albumin, the cells were incubated with anti-β-catenin (Proteintech, Wuhan, China) at 4 °C overnight, followed by incubation with FITC-conjugated secondary antibody at 37 °C for 1 h. The cell nuclei were stained with DAPI reagent. The nuclear translocation of β-catenin was observed under a fluorescence microscope. Three fields in each well were randomly selected to determine the average ratio of FITC^+^/DAPI^+^ cells.

### *In vitro* coculture and osteoclast differentiation

Bone marrow cells were isolated from the tibias and femurs of C57BL/6J mice aged 6–8 weeks and seeded onto a 48-well plate at a density of 4 × 10^5^ cells/well. The cells were cultured for 3 days in the presence of 20 ng/mL CSF1 in α-MEM containing 10% fetal bovine serum. Nonadherent cells were removed, and the medium was refreshed. In parallel, wild-type BMSCs at passage 3 were infected with lentivirus expressing NKD2 or the vector. Upon reaching 80% confluence, the BMSCs were digested and added to the plate of bone marrow cells at a density of 3000 cells/well. The cocultures were maintained in the presence of 20 ng/mL CSF1 and 10^−7^ mol/L 1,25(OH)_2_D_3_ to allow osteoclast differentiation. The medium was refreshed every 3 days. Seven days after induction, the cells were stained for tartrate resistant acid phosphatase (TRAP) using a kit from Sigma–Aldrich (St. Louis, MO, USA). TRAP-positive multinucleated osteoclasts (≥3 nuclei) were counted.

### *In vivo* transplantation of primary BMSCs

Primary BMSCs were isolated from C57BL/6J mice aged 4–6 weeks. Cells at passage 3 were infected with lentivirus expressing NKD2 or control virus for 24 h. Female C57BL/6J mice were purchased from SPF Biotechnology (Beijing, China) and maintained in a specific pathogen-free animal facility with free access to water and food. Eleven-week-old mice were randomly allocated into four groups (*n* = 8 per group): Sham/control group, Sham/NKD2 group, OVX/control group, and OVX/NKD2 group. The mice received intratibial transplantation of the aforementioned NKD2-overexpressing cells or control cells twice following a procedure as previously described.^26^ Three days after transplantation, the mice received bilateral ovariectomy or sham operation. One month after the surgery, the mice received a second transplantation. Two months after surgery, the mice were euthanized, and the transplanted bones were collected. Histological and immunohistochemical staining were performed, and μCT analyses were performed on the samples. Animal protocols were approved by the Animal Ethics Committee of Tianjin Medical University Chu Hsien-I Memorial Hospital.

### *Ex vivo* osteoclast differentiation

Two weeks following surgery, bone marrow cells were isolated from transplanted tibias and plated on a 48-well plate at a density of 4 × 10^5^ cells/well. The cells were grown in the presence of 20 ng/mL CSF1 in α-MEM containing 10% fetal bovine serum for 3 days. Nonadherent cells were removed, and adherent cells continued to be cultured in fresh medium in the presence of 20 ng/mL CSF1 and 10^−7^ mol/L 1,25(OH)_2_D_3_ to allow osteoclast differentiation. Five days following induction, total RNA and protein were extracted, and the expression levels of osteoclastogenic genes were measured using RT-qPCR and western blotting. Seven days following induction, the cells were fixed and stained for TRAP.

#### μCT analyses

μCT measurement was carried out using a Vivo-CT80 μCT scanner (Scanco Medical, Switzerland). Briefly, tibias were scanned at high resolution with a voxel size of 10.4 μm. Parameters of the trabecular and cortical bones were analyzed in a region of interest beginning 0.2 mm below the growth plate and extending 1.0 mm distally.

### Histological and immunohistochemical (IHC) staining

Transplanted tibias were fixed with 10% formalin for 3 days, decalcified in 14% EDTA (pH 7.4) for 21 days, and then dehydrated in a graded ethanol series (70%–100%). The samples were embedded in paraffin and cut into 4-μm-thickness serial mid-sagittal sections for histological and IHC analyses. Bone marrow fat was quantified by measuring the numbers and areas of adipocytes on the sections stained with hematoxylin and eosin. TRAP staining was performed on the sections to identify osteoclasts using a staining solution consisting of acetate buffer (0.1 M, pH 5.0), naphthol AS-MX phosphate (0.1 mg/mL), fast red violet LB salt (0.6 mg/mL), and sodium tartrate (0.05 M). The region of interest begins 0.2 mm below the growth plate and extends 1.0 mm distally.

ALP IHC staining was performed to identify and quantify the trabecular osteoblasts on the slides. Briefly, the sections were baked at 60 °C overnight and then deparaffinized and rehydrated. Antigen retrieval was performed by incubating the sections with 0.075% trypsin at 37 °C for 15 min. Subsequently, the sections were incubated at 4 °C with an anti-ALP antibody (ab108337, Abcam, Cambridge, MA) overnight and then with an HRP-conjugated secondary antibody for 2 h. The sections were developed in 3,3′-diaminobenzidine solution for 5 min to visualize ALP-positive cells. The nuclei were counterstained with hematoxylin for 3 s. IHC staining of TSC1 and RANKL was also performed using anti-TSC1 (29906-1-AP, Proteintech, Wuhan, China) and anti-RANKL (WL00285, Wanleibio, Shenyang, China) antibodies.

### Statistical analysis

The data were expressed as mean ± standard deviation. Student's *t*-test was applied for comparisons between two groups. One-way or two-way ANOVA was applied for comparisons among three or more groups. Post hoc comparisons were performed using Dunnett's test following one-way ANOVA or using Tukey's test following two-way ANOVA. *P* values < 0.05 were considered statistically significant.

## Results

### NKD2 was regulated during the differentiation of mesenchymal stem/progenitor cells

NKD2 mRNA levels in various tissues were evaluated by RT-qPCR. It was most abundant in bone, moderate in skeletal muscle, and low in colon, brain, heart, liver, and spleen (Fig. S1A). The expression pattern of NKD2 was investigated during the differentiation of primary BMSCs and established cell lines (Figs. S1B–E). Overall, NKD2 was increased in both osteogenic and adipogenic differentiation at most indicated time points, suggesting that NKD2 may play a role in mesenchymal cell differentiation.

### NKD2 promoted osteoblast differentiation

**Figure 1:**
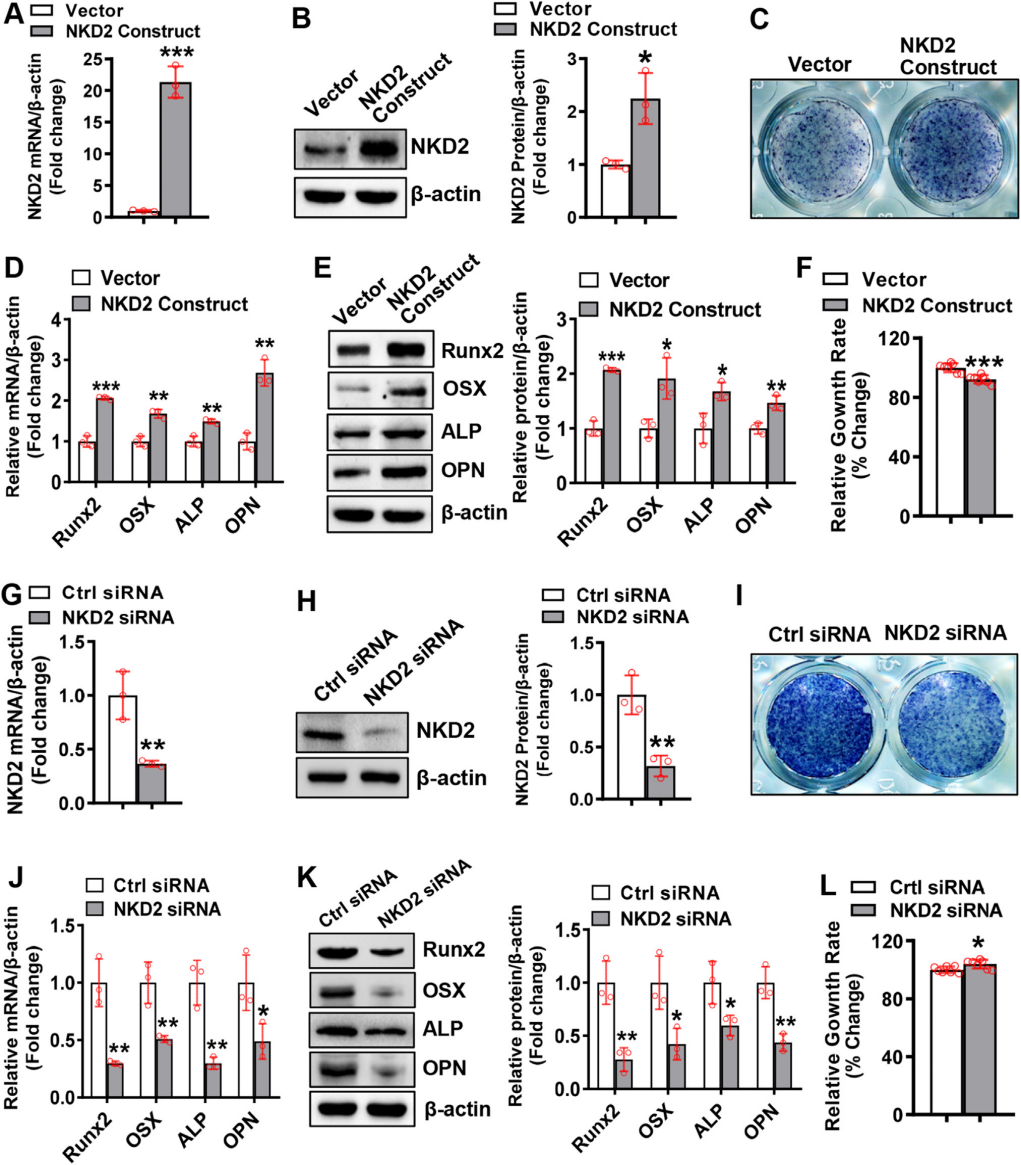
Reverse transcription and quantitative PCR and western blotting were performed to evaluate the mRNA and protein levels of NKD2 in ST2 cells 48 h after transfection with the NKD2 expression construct **(A, B)** or siRNAs **(G, H)**. The transfected cells were grown in an osteogenic medium to allow osteoblast differentiation. ALP staining was applied to differentiated osteoblasts **(C, I)**. The mRNA **(D, J)** and protein **(E, K)** levels of osteogenic factors were determined 3 days following osteogenic treatment. A CCK-8 assay was carried out to measure the growth rate of ST2 following NKD2 overexpression or silencing **(F, L)**. The values were expressed as mean ± standard deviation; *n* = 3 in (A, B, D, E, G, H, J, K); *n* = 7 in (F, L). ∗*P* < 0.05, ∗∗*P* < 0.01, ∗∗∗*P* < 0.001 *vs.* vector or control siRNA. NKD2, naked cuticle homolog 2.

We performed gain-of-function and loss-of-function experiments to clarify the function of NKD2 in osteogenesis. Transfection of the NKD2 expression construct drastically induced mRNA and protein expression of NKD2 in ST2 cells (Fig. 1A, B). Following osteogenic treatment, the overexpression of NKD2 promoted osteogenic differentiation of ST2 cells, as indicated by enhanced ALP staining when compared with control cells (Fig. 1C). Accordingly, NKD2 overexpression drastically induced mRNA and protein expression of osteogenic factors, *e.g.*, runt-related transcription factor-2, osterix, ALP, and osteopontin, at 3 and 5 days after osteogenic treatment (Fig. 1D, E; Fig. S2A and B). The CCK8 assay showed that NKD2 slightly inhibited the cell growth rate of ST2 by 8% (Fig. 1F). Similarly, overexpressing NKD2 in primary BMSCs drastically enhanced osteoblast differentiation and up-regulated the expression of osteogenic factors (Figs. S3A–F).

Conversely, transfection of NKD2 siRNA knocked down NKD2 mRNA and protein in ST2 cells (Fig. 1G, H). In the presence of osteogenic agents, the depletion of NKD2 inhibited osteogenic differentiation of ST2 cells, as indicated by weakened ALP staining (Fig. 1I). Accordingly, NKD2 silencing drastically reduced the mRNA and protein levels of osteogenic factors at 3 and 5 days after osteogenic treatment (Fig. 1J, K; Fig. S2C, D). Furthermore, NKD2 siRNA slightly increased the cell growth rate of ST2 by 4% (Fig. 1L).

### NKD2 inhibited adipocyte differentiation

**Figure 2:**
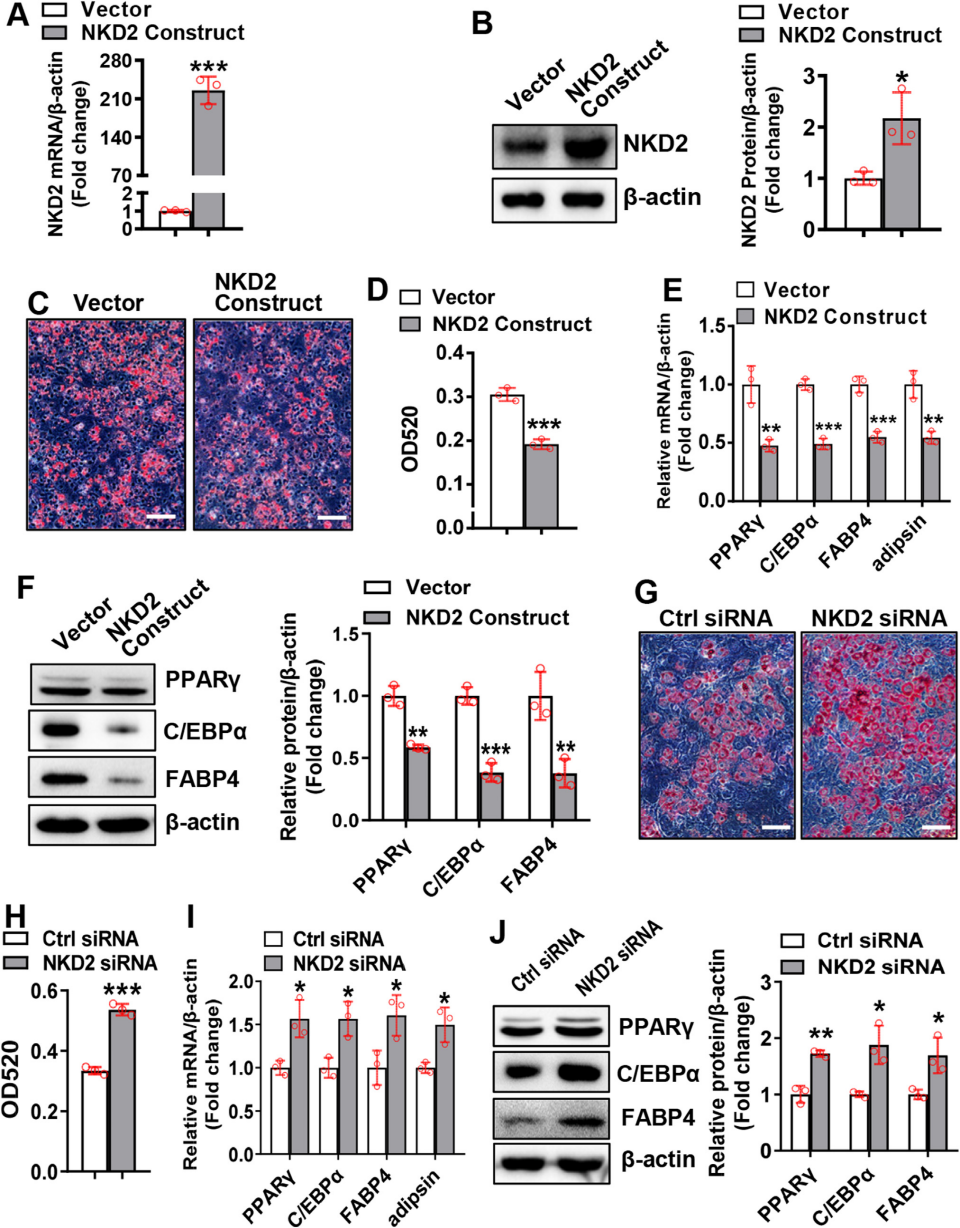
NKD2 inhibited adipocyte differentiation. Reverse transcription and quantitative PCR and western blotting were performed to evaluate NKD2 mRNA and protein levels in C3H10T1/2 cells 48 h after transfection with the NKD2 expression construct or vector **(A, B)**. The effects of NKD2 overexpression **(C–F)** or knockdown **(G**–**J)** on adipocyte differentiation from C3H10T1/2 **(C–F)** or ST2 **(G**–**J)** cells were evaluated. Differentiated adipocytes were stained with oil red O **(C, G)**. The staining intensity was evaluated by extracting the stain from the cells and measuring OD520 **(D, H)**. The mRNA **(E, I)** and protein **(F, J)** levels of adipogenic factors were measured 3 days following adipogenic treatment. Image scale in (C, G): 100 μm. The values were expressed as mean ± standard deviation; *n* = 3. ∗*P* < 0.05, ∗∗*P* < 0.01, ∗∗∗*P* < 0.001 *vs.* vector or control siRNA. NKD2, naked cuticle homolog 2.

We performed gain-of-function and loss-of-function experiments to clarify the function of NKD2 in adipogenesis. NKD2 was successfully overexpressed in C3H10T1/2 cells (Fig. 2A, B). Under adipogenic conditions, overexpression of NKD2 inhibited adipocyte differentiation from C3H10T1/2 cells, as indicated by the decreased intensity of oil red O staining (Fig. 2C, D). Accordingly, NKD2 overexpression drastically suppressed the mRNA and protein expression of adipogenic factors, *e.g.*, peroxisome proliferator-activated receptor γ, CCAAT/enhancer binding protein α, fatty acid binding protein 4, and adipsin, at 2 and 3 days after adipogenic treatment (Fig. S4A, B; Fig. 2E, F). Similarly, overexpressing NKD2 in primary BMSCs drastically suppressed adipocyte formation and down-regulated adipogenic factors (Fig. S5).

Conversely, following adipogenic treatment, silencing of NKD2 promoted adipocyte differentiation from ST2 cells, as indicated by enhanced oil red O staining intensity (Fig. 2G, H). Accordingly, NKD2 silencing drastically up-regulated adipogenic factors at 2 and 3 days after adipogenic treatment (Fig. 2I, J; Fig. S4C, D).

### NKD2 differentially regulated Wnt/β-catenin signaling in undifferentiated mesenchymal cells and those undergoing differentiation

**Figure 3:**
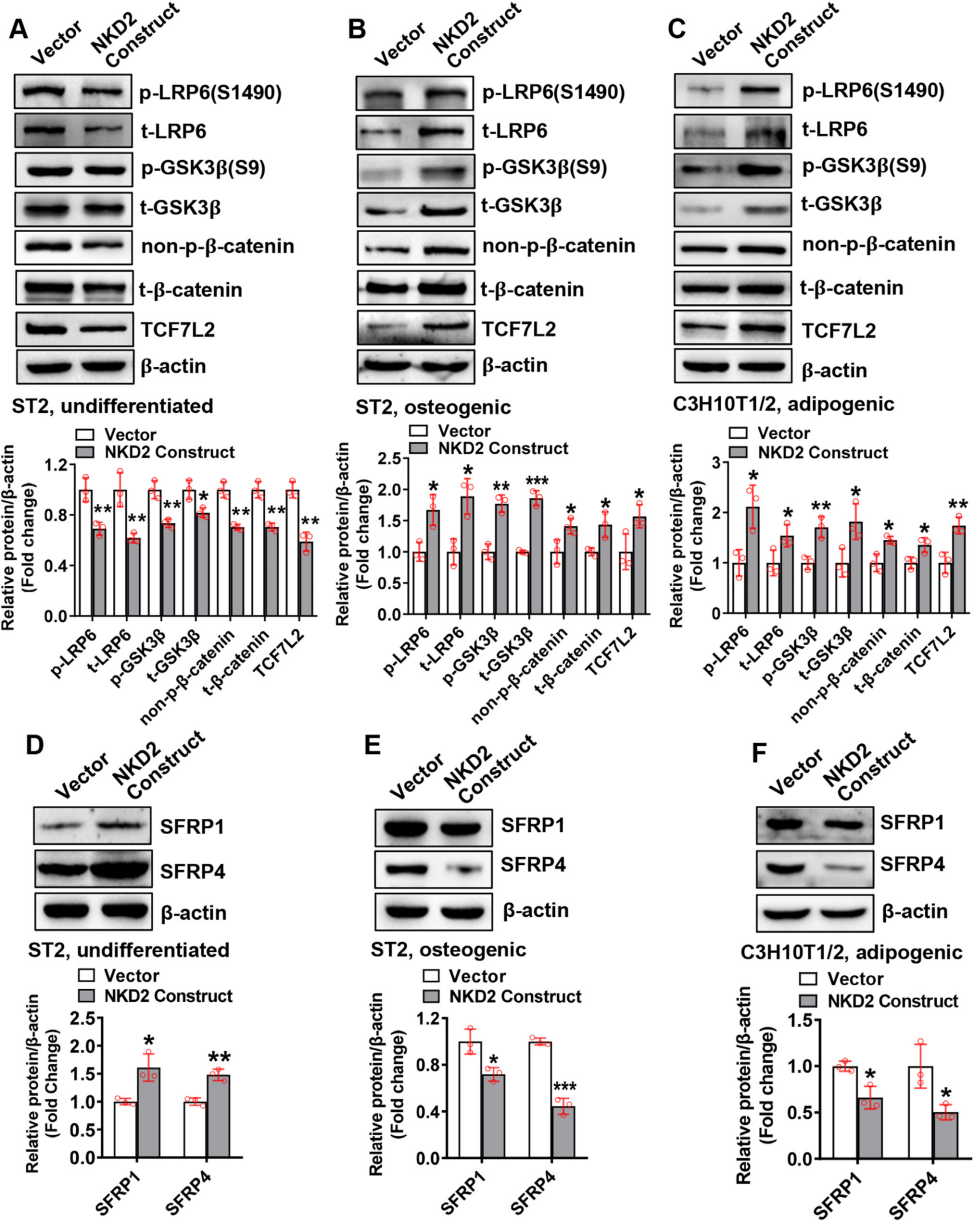
NKD2 differentially regulated Wnt/β-catenin signaling in undifferentiated mesenchymal cells and those undergoing differentiation. ST2 **(A, B)** or C3H10T1/2 **(C)** cells were transfected with the NKD2 expression construct or vector for 4 h and then cultured in common medium **(A, D)**, osteogenic medium **(B, E)**, or adipogenic medium **(C, F)** for 72 h. Western blotting was performed to measure the protein levels of the major components in canonical Wnt signaling pathway **(A**–**C)**. The protein levels of secreted frizzled related protein 1 (SFRP1) and secreted frizzled related protein 4 (SFRP4) were also measured **(D**–**F)**. The values were expressed as mean ± standard deviation; *n* = 3. ∗*P* < 0.05, ∗∗*P* < 0.01, ∗∗∗*P* < 0.001 *vs.* vector. NKD2, naked cuticle homolog 2.

NKD2 inhibits Wnt/β-catenin signaling in tumorigenesis.^21^ We investigated whether NKD2-regulated cell differentiation involves the Wnt/β-catenin pathway. In undifferentiated ST2 cells, NKD2 overexpression significantly down-regulated phospho-low-density lipoprotein receptor-related protein 6 (S1490), phospho-glycogen synthase kinase 3β (S9), non-phospho-β-catenin, and transcription factor 7 like 2 (Fig. 3A). However, in ST2 cells differentiating toward osteoblasts and in C3H10T1/2 cells differentiating toward adipocytes, NKD2 overexpression increased these proteins (Fig. 3B, C). Consistently, immunofluorescence staining revealed that NKD2 overexpression suppressed the nuclear translocation of β-catenin in undifferentiated cells (Fig. S6A), while in cells differentiating toward osteoblasts and adipocytes, NKD2 overexpression stimulated the nuclear translocation of β-catenin (Fig. S6B, C). Moreover, NKD2 overexpression down-regulated cyclin D1 expression in undifferentiated ST2 cells (Fig. S6D), while in cells differentiating toward osteoblasts and adipocytes, NKD2 overexpression up-regulated cyclin D1 (Fig. S6E, F). We further investigated how NKD2 affects Wnt/β-catenin signaling. In undifferentiated ST2 cells, NKD2 overexpression increased secreted frizzled related protein 1 (SFRP1) and secreted frizzled related protein 4 (SFRP4) protein levels (Fig. 3D), while in cells undergoing osteogenic and adipogenic differentiation, NKD2 overexpression decreased SFRP1 and SFRP4 protein levels (Fig. 3E, F).

Furthermore, we examined the expression pattern of these key players in primary BMSCs during differentiation (Fig. S7). SFRP1 was up-regulated at some time points during osteogenic and adipogenic differentiation (Fig. S7B, H). SFRP4 was slightly up-regulated on day 1 and down-regulated by the end of osteogenic induction (day 13). In contrast, it was transiently up-regulated on day 1 during adipogenic differentiation (Fig. S7C, I).

### NKD2 differentially regulated DVL1 in undifferentiated and differentiating cells

It was reported that NKD2 antagonizes Wnt/β-catenin signaling by enhancing the degradation of DVL1 at the plasma membrane.^27^ In our study, we found that DVL1 was down-regulated at the early stage (day 4) and up-regulated at the late stage (day 13) of osteogenic differentiation. In contrast, it was up-regulated at the early stage (day 1) and down-regulated at the late stage (days 7 and 9) of adipogenic differentiation (Fig. S7D, J).

**Figure 4:**
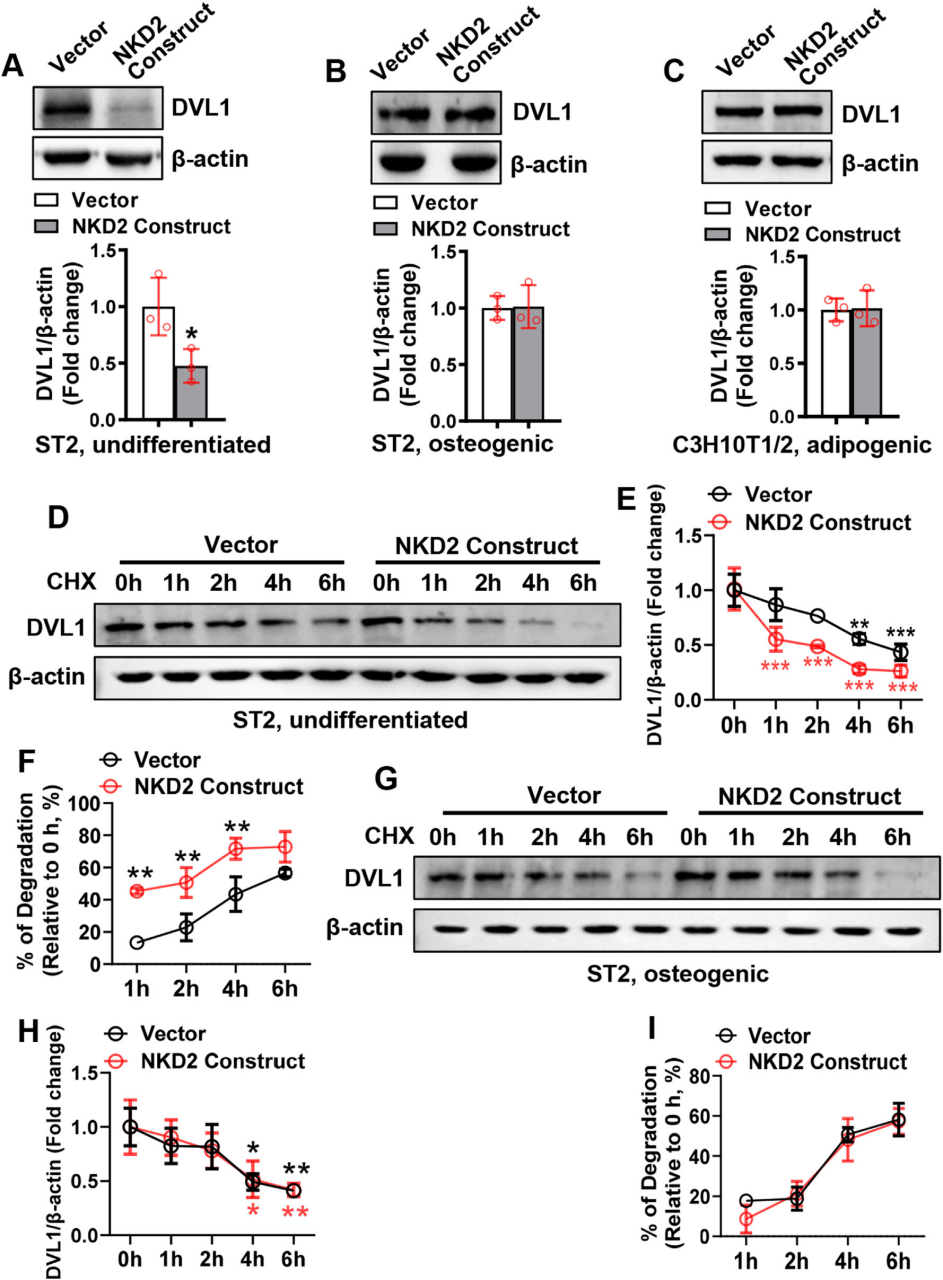
NKD2 differentially regulated DVL1 in undifferentiated mesenchymal cells and those undergoing differentiation. ST2 **(A, B)** or C3H10T1/2 cells **(C)** were transfected with the NKD2 expression construct or vector for 4 h and then cultured in common medium **(A)**, osteogenic medium **(B)**, or adipogenic medium **(C)** for 72 h. The protein level of DVL1 was evaluated using western blotting. ST2 cells were transfected with the NKD2 expression construct or vector for 4 h and then cultured in a common medium for 24 h **(D)** or in an osteogenic medium for 48 h **(G)**, followed by cycloheximide treatment. Then, the protein level of DVL1 was examined at the indicated time points **(E, H)**. The percentage of DVL1 degradation relative to the start point (0 h) was calculated **(F, I)**. The values were expressed as mean ± standard deviation; *n* = 3. (A–C, F, I), ∗*P* < 0.05, ∗∗*P* < 0.01 *vs.* vector; (E, H), ∗*P* < 0.05, ∗∗*P* < 0.01, ∗∗∗*P* < 0.001 *vs.* 0 h. NKD2, naked cuticle homolog 2; DVL1, Dishevelled 1.

NKD2 overexpression reduced DVL1 protein level in undifferentiated ST2 cells 72 h after transfection (Fig. 4A) but not in ST2 cells undergoing osteogenic differentiation or C3H10T1/2 cells undergoing adipogenic differentiation (Fig. 4B, C). Protein degradation assays were performed in undifferentiated cells 24 h after transfection. It showed that significant degradation of DVL1 occurred at 1 h through 6 h following cycloheximide treatment in NKD2-overexpressing cells, much earlier than in vector-transfected cells (4–6 h) (Fig. 4D, E). The percentage of DVL1 degradation at 1, 2, and 4 h relative to 0 h was higher in NKD2-overexpressing cells than in vector-transfected cells (Fig. 4F). In cells undergoing osteogenic differentiation, significant degradation occurred at 4–6 h in both groups (Fig. 4G, H). The percentage of DVL1 degradation at each indicated time point relative to 0 h was unchanged in NKD2-overexpressing cells versus vector-transfected cells (Fig. 4I).

### Silencing β-catenin attenuated NKD2-induced deregulation of osteogenic and adipogenic differentiation

To further clarify whether NKD2-regulated cell differentiation is dependent on Wnt/β-catenin signaling, we performed a cotransfection experiment. The data showed that under osteogenic induction, cotransfection with the NKD2 expression construct and control siRNA enhanced ALP staining (Fig. S8A) and up-regulated osteogenic factors at 3 days and 5 days (Fig. S8B, 8C, 9A, 9B) after osteogenic induction compared with cotransfection with vector and control siRNA. This stimulatory effect of NKD2 was attenuated when the cells were cotransfected with the NKD2 expression construct and β-catenin siRNA (Figs. S8A–C; Fig. S9A, B). In contrast, under adipogenic induction, the inhibitory effect of NKD2 on adipocyte differentiation was attenuated when the cells were cotransfected with the NKD2 expression construct and β-catenin siRNA (Figs. S8D–G; Fig. S9C, D).

### NKD2 interacted with TSC1 and suppressed the mechanistic target of rapamycin kinase complex 1 (mTORC1) signaling

**Figure 5:**
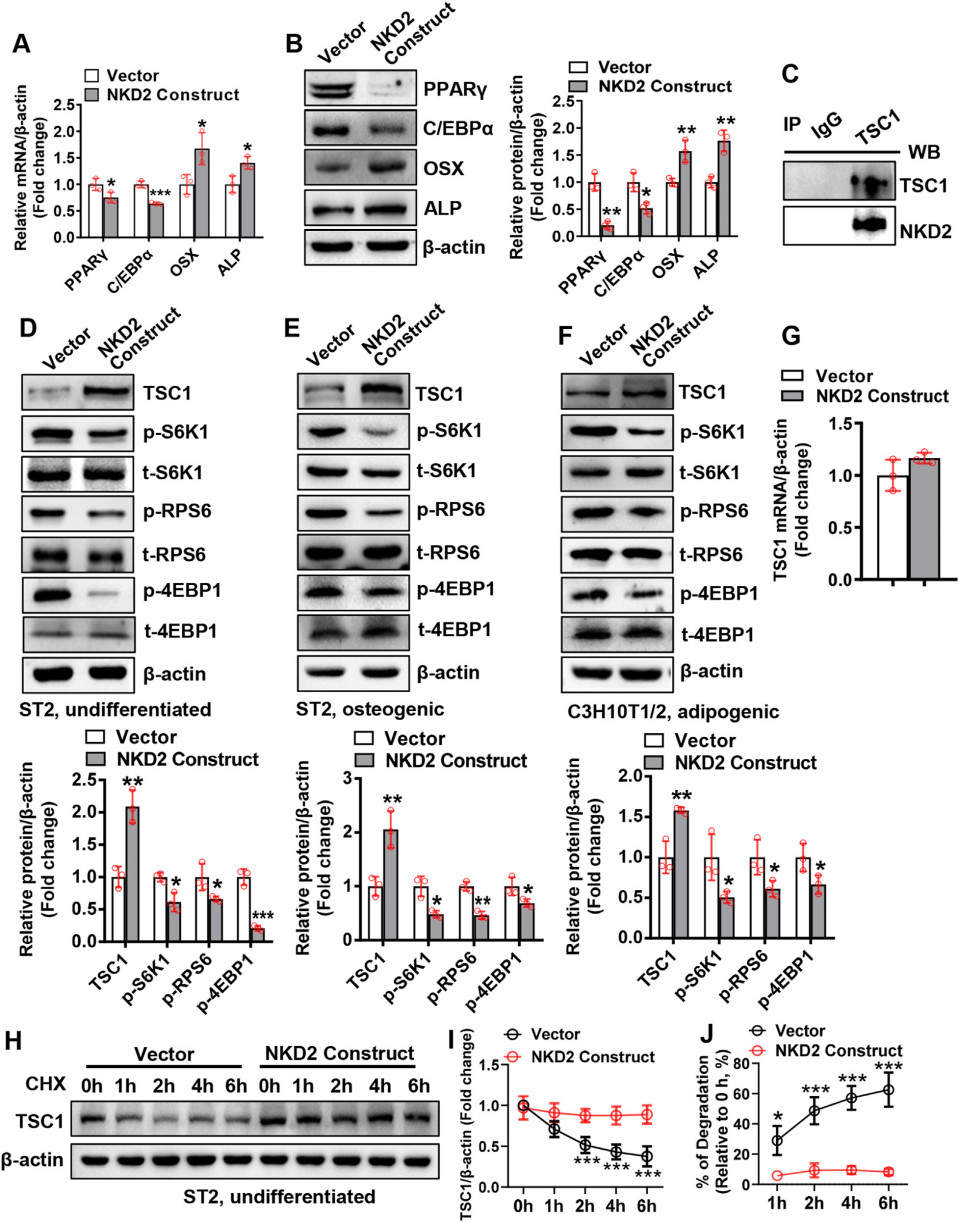
NKD2 interacted with TSC1 and suppressed mTORC1 signaling. The mRNA **(A)** and protein **(B)** levels of osteogenic factors and adipogenic factors were measured in undifferentiated ST2 cells 72 h after transfection. A coimmunoprecipitation experiment was performed to examine the interaction between NKD2 and TSC1 in ST2 48 h following transfection of NKD2 expression construct **(C)**. The protein levels of TSC1 and other major components in mTORC1 signaling pathway were measured in undifferentiated ST2 cells (transfected for 4 h followed by 72 h of culture) **(D)**, in differentiating ST2 cells (transfected for 4 h followed by 72 h of osteogenic treatment) **(E)**, or in differentiating C3H10T1/2 cells (transfected for 4 h followed by 72 h of adipogenic treatment) **(F)**. The mRNA level of TSC1 was measured in undifferentiated ST2 cells **(G)**. ST2 cells were transfected with the NKD2 expression construct or vector for 4 h and then cultured in a common medium for 24 h, followed by cycloheximide treatment **(H)**. The protein level of TSC1 was examined at the indicated time points **(I)**. The percentage of TSC1 degradation relative to the start point (0 h) was calculated **(J)**. The values were expressed as mean ± standard deviation; *n* = 3. ∗*P* < 0.05, ∗∗*P* < 0.01, ∗∗∗*P* < 0.001 *vs.* vector. NKD2, naked cuticle homolog 2; TSC1, tuberous sclerosis complex subunit 1; mTORC1mechanistic target of rapamycin complex 1.

Since NKD2 inhibited canonical Wnt signaling in undifferentiated cells, we investigated how NKD2 affects osteogenic and adipogenic factors in the undifferentiated state. The data showed that NKD2 up-regulated osteogenic factors and down-regulated adipogenic factors (Fig. 5A, B). This suggests that there exist other signaling pathways involved in the differentiation of mesenchymal stem/progenitor cells. Bioinformatics analysis predicted TSC1 as a binding partner of NKD2 (https://thebiogrid.org/124517/summary/homo-sapiens/nkd2.html). TSC1 was up-regulated at the middle stage of osteogenic differentiation from BMSCs (days 4 and 7). During adipogenic differentiation, it was up-regulated at the early stage (day 1) and down-regulated at the late stage (day 7) (Fig. S7E, K). The coimmunoprecipitation experiment confirmed that NKD2 was able to bind with TSC1 (Fig. 5C). Furthermore, NKD2 overexpression up-regulated TSC1 protein and down-regulated other major components of mTORC1 signaling such as phospho-ribosomal protein S6 kinase polypeptide 1, phospho-ribosomal protein S6, and phospho-eukaryotic translation initiation factor 4E binding protein 1, in ST2 cells that were either undifferentiated or differentiating toward osteoblasts, as well as in C3H10T1/2 cells differentiating toward adipocytes (Fig. 5D–F). In contrast, NKD2 overexpression did not affect TSC1 mRNA expression in undifferentiated ST2 cells (Fig. 5G). Protein degradation assays showed that significant degradation of TSC1 occurred at 2 h through 6 h following cycloheximide treatment in vector-transfected cells, while degradation did not occur in NKD2-overexpressing cells (Fig. 5H–J).

### Silencing TSC1 attenuated NKD2-induced deregulation of osteogenic and adipogenic differentiation

We then tried to clarify whether NKD2-regulated cell differentiation is dependent on TSC1/mTORC1 signaling. The data for the cotransfection experiment showed that under osteogenic induction, cotransfection with the NKD2 expression construct and control siRNA enhanced ALP staining (Fig. S10A) and up-regulated osteogenic factors at 3 days and 5 days after induction (Figs. S10B, C, 11A, B) compared with transfection with vector and control siRNA. This stimulatory effect of NKD2 was attenuated when the cells were cotransfected with the NKD2 expression construct and TSC1 siRNA (Fig. S10A–C, 11A, B). In contrast, under adipogenic induction, the inhibitory effect of NKD2 on adipocyte differentiation was attenuated when the cells were cotransfected with the NKD2 expression construct and TSC1 siRNA (Fig. S10D–G, 11C, D).

### Depletion of TSC1 inactivated NKD2-regulated Wnt/β-catenin signaling in undifferentiated and differentiating cells

We then performed a cotransfection experiment to clarify whether there is an interplay between TSC1/mTORC1 and Wnt/β-catenin signaling. The data showed that in undifferentiated cells and those cells differentiating toward osteoblasts and adipocytes, regardless of whether NKD2 was overexpressed, depletion of TSC1 down-regulated phospho-glycogen synthase kinase 3β (S9) and non-phospho-β-catenin, thus inactivating Wnt/β-catenin signaling (Figs. S12A–C).

### Transplantation of NKD2-overexpressing BMSCs partially prevented cancellous bone loss in ovariectomized mice

**Figure 6:**
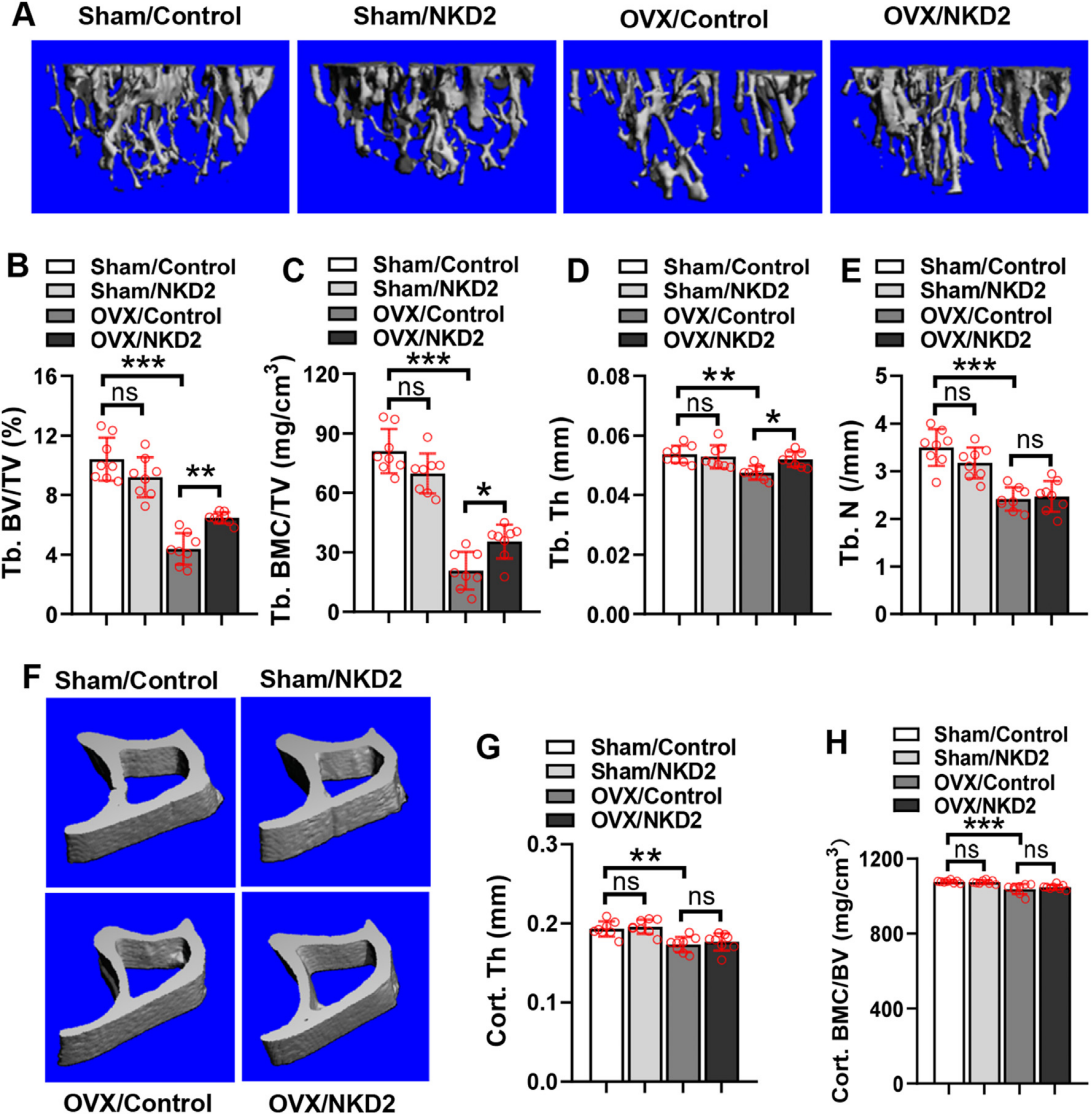
Transplantation of NKD2-overexpressing BMSCs partially prevented cancellous bone loss in ovariectomized mice. μCT analysis of bone mass in the proximal metaphysis of transplanted tibias was performed, and the reconstruction images for cancellous bone **(A)** and cortical bone **(F)** are shown. Histomorphometric parameters were analyzed in the mice for cancellous bone **(B**–**E)** and cortical bone **(G, H)**. The values were expressed as mean ± standard deviation; *n* = 8. ∗*P* < 0.05, ∗∗*P* < 0.01, ∗∗∗*P* < 0.001. ns, no significance; NKD2, naked cuticle homolog 2; BMSCs, bone marrow mesenchymal stem cells.

We next investigated whether NKD2 modulates *in vivo* differentiation of BMSCs and bone homeostasis in mice. μCT measurements showed that 2 months after surgery, histomorphometric parameters reflecting the quality of cancellous bone including trabecular bone volume/total volume, trabecular bone mineral content/total volume, trabecular thickness, and trabecular number, and parameters reflecting the quality of cortical bone including cortical thickness and cortical bone mineral content/bone volume were reduced in OVX/Control group compared with Sham/Control group (Fig. 6A–H). Following transplantation, these parameters were not altered in Sham/NKD2 group versus Sham/Control group (Fig. 6A–H). In contrast, the former three parameters were increased, while latter three parameters were unchanged in OVX/NKD2 group versus OVX/Control group (Fig. 6A–H).

### Transplantation of NKD2-overexpressing BMSCs increased osteoblasts and reduced adipocytes in OVX mice

**Figure 7:**
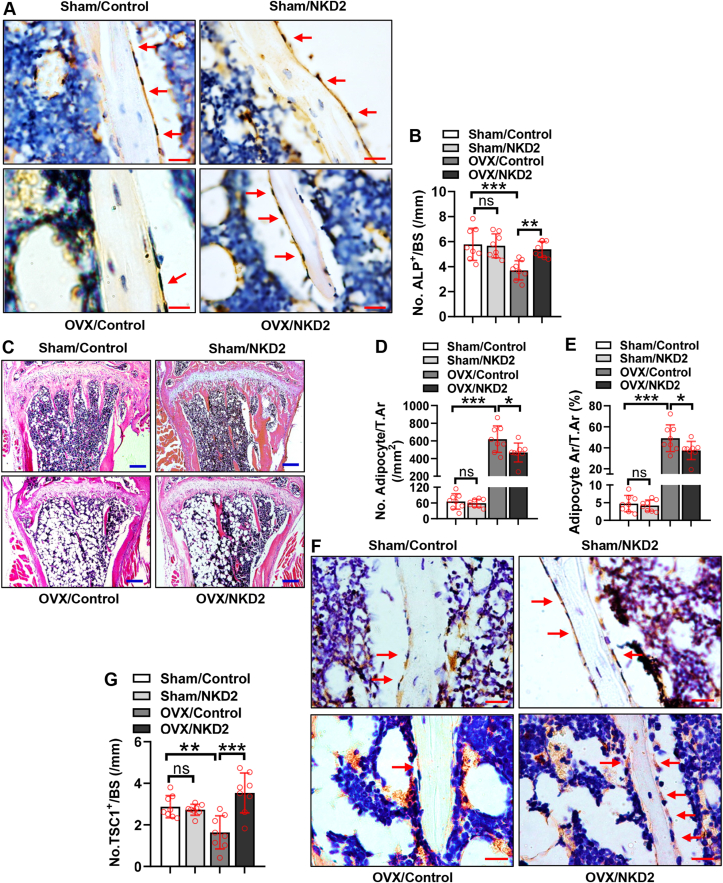
NKD2 in BMSCs facilitated osteoblast differentiation and suppressed adipocyte formation in OVX mice. Representative images of immunohistochemical staining of ALP are shown **(A)**. The number of osteoblasts was quantified **(B)**. Representative images of hematoxylin and eosin staining are shown **(C)**. The number **(D)** and area **(E)** of adipocytes were quantified. Representative images of immunohistochemical staining of TSC1 are shown **(F)**. The number of TSC1-positive cells on the trabeculae was counted **(G)**. Scale bar in (A, F): 20 μm. Scale bar in (C): 200 μm. The values were expressed as mean ± standard deviation; *n* = 8. ∗*P* < 0.05, ∗∗*P* < 0.01, ∗∗∗*P* < 0.001. ns, no significance; NKD2, naked cuticle homolog 2; BMSCs, bone marrow mesenchymal stem cells.

We investigated the cellular basis for the *in vivo* effects of NKD2-overexpressing BMSCs. ALP-positive osteoblasts were drastically reduced on the trabeculae, while adipocytes (number and area) were increased in the marrow of OVX/Control mice compared with Sham/Control mice (Fig. 7A–E). Following transplantation, the number of osteoblasts was not changed in Sham/NKD2 group compared with Sham/Control group, nor were the number and area of adipocytes (Fig. 7A–E). However, osteoblasts were significantly increased, while adipocytes (number and area) were reduced in OVX/NKD2 mice compared with OVX/Control mice (Fig. 7A–E). Moreover, IHC staining revealed that the number of TSC1-positive cells on the trabeculae was not changed in Sham/NKD2 group, but was significantly reduced in OVX/Control group compared with Sham/Control group (Fig. 7F, G). Of note, they were increased in OVX/NKD2 group compared with OVX/Control group (Fig. 7F, G).

### NKD2 in mesenchymal cells regulated osteoclast differentiation by a RANKL-dependent mechanism

**Figure 8:**
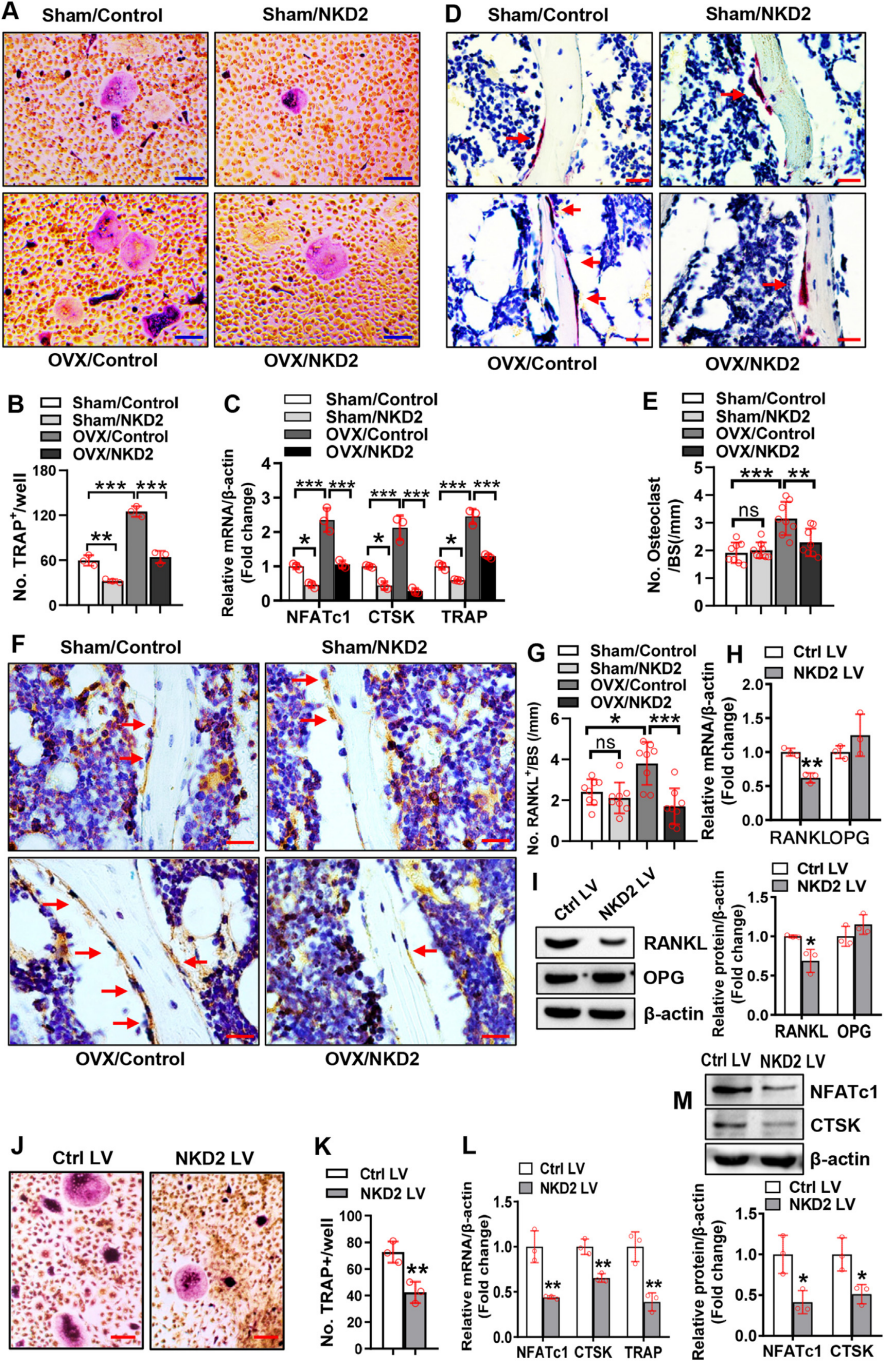
NKD2 in BMSCs regulated osteoclast differentiation by a RANKL-dependent mechanism. Bone marrow cells were isolated from mice 2 weeks after surgery, cultured, and induced to allow osteoclast differentiation. TRAP staining **(A)** was performed 7 days after induction, and osteoclasts were counted **(B)**. The mRNA expression levels of osteoclastogenic factors were measured 5 days after induction **(C)**. Transplanted tibia sections were stained for TRAP **(D)**, and osteoclasts were counted **(E)**. Representative images of immunohistochemical staining of RANKL are shown **(F)**, and RANKL-positive cells on the trabeculae were counted **(G)**. The mRNA and protein levels of RANKL and osteoprotegerin (OPG) were measured in BMSCs 48 h after infection with NKD2 expression LV **(H, I)**. Wild-type marrow osteoclast precursors were cocultured with NKD2-overexpressing BMSCs or control BMSCs and induced to allow osteoclast differentiation. TRAP staining **(J)** was performed 7 days after induction, and osteoclasts were counted **(K)**. The mRNA and protein levels of osteoclastogenic factors were measured 5 days after induction **(L, M)**. Scale bar in (A, J): 100 μm; Scale bar in (D, F): 20 μm. The values were expressed as mean ± standard deviation; *n* = 3 in (B, C, H, I, K–M); *n* = 8 in (E, G). ∗*P* < 0.05, ∗∗*P* < 0.01, ∗∗∗*P* < 0.001. ns, no significance; TRAP, tartrate resistant acid phosphatase; NKD2, naked cuticle homolog 2; BMSCs, bone marrow mesenchymal stem cells; RANKL, receptor activator of nuclear factor-κB ligand.

We further investigated whether and how NKD2 in mesenchymal stem/progenitor cells regulates osteoclast differentiation at both *in vitro* and *in vivo* levels. Two weeks after surgery, osteoclast formation from the marrow cells of OVX/Control mice was enhanced compared with that of Sham/Control mice. The transplantation of NKD2-overexpressing BMSCs relieved osteoclast formation both in the marrow cells from Sham/NKD2 mice compared with those from Sham/Control mice and in the marrow cells from OVX/NKD2 mice when compared with those from OVX/Control mice (Fig. 8A, B). Similar changes in the mRNA expression of osteoclastogenic factors, such as nuclear factor of activated T cells 1, cathepsin K, and TRAP, were found among the groups (Fig. 8C). Furthermore, 2 months after surgery, TRAP-positive osteoclasts were increased in OVX/Control mice compared with Sham/Control mice (Fig. 8D, E). Following transplantation, the number of osteoclasts was unchanged in Sham/NKD2 group versus Sham/Control group. In contrast, osteoclasts were reduced in OVX/NKD2 mice versus OVX/Control mice (Fig. 8D, E).

For mechanistic investigation, IHC staining revealed that the number of RANKL-positive cells on the trabeculae was unchanged in Sham/NKD2 group, but increased in OVX/Control mice versus Sham/Control mice (Fig. 8F, G). In contrast, they were significantly reduced in OVX/NKD2 mice compared with OVX/Control mice (Fig. 8F, G). Consistently, NKD2 overexpression in BMSCs significantly down-regulated RANKL expression but had no effect on osteoprotegerin expression (Fig. 8H, I). In ST2 cells, we also observed that NKD2 overexpression down-regulated RANKL mRNA and protein, but it had no effect on osteoprotegerin expression (Fig. S13A, B). Silencing β-catenin relieved the down-regulation of RANKL by NKD2 (Fig. S13A, B). Furthermore, fewer osteoclasts formed in the cocultures of NKD2-overexpressing BMSCs and marrow osteoclast precursors than in the cocultures of control BMSCs and osteoclast precursors (Fig. 8J, K). Accordingly, the cocultures of NKD2-overexpressing BMSCs and marrow osteoclast precursors had lower expression levels of osteoclastogenic factors (Fig. 8L, M).

## Discussion

Although the role of NKD2 in the *in vitro* osteogenic differentiation of dental follicle cells has ever been documented,^24^ it remains to be clarified whether and how NKD2 in mesenchymal stem/progenitor cells plays a role in the *in vitro* and *in vivo* differentiation of osteoblasts and adipocytes and in bone homeostasis. Moreover, it is of equal interest to understand whether and how NKD2 regulates osteoclast formation.

Among the variety of mouse tissues we examined, NKD2 was most abundant in bone. Moreover, NKD2 expression was elevated during both osteoblast differentiation and adipocyte formation, suggesting that NKD2 may have a role during the differentiation of mesenchymal stem/progenitor cells. Functional experiments revealed that NKD2 slightly inhibited the growth rate of ST2 cells, which coincides with the previously identified role of NKD2 in suppressing tumor cell growth.^21^, ^22^, ^23^ Regarding its role in differentiation, NKD2 promoted osteoblast differentiation and conversely suppressed adipocyte differentiation. Because of the importance of Wnt/β-catenin signaling for bone and the reported function of NKD2 in affecting DVL1 stability,^28^^,^^29^ we investigated whether NKD2 modulates Wnt/β-catenin signaling in mesenchymal stem/progenitor cells. Of interest, we found that NKD2 functioned differentially in undifferentiated and differentiating cells. Consistent with previously reported findings,^19^^,^^27^ NKD2 suppressed canonical Wnt signaling in undifferentiated cells. Notably, a stimulatory effect was observed in the cells differentiating toward osteoblasts or adipocytes. Obviously, NKD2 exerts opposite effects on canonical Wnt signaling depending on differentiation or not. This was never reported in the study of Chen et al, which focused on NKD2 regulation of dental follicle stem/progenitor cell differentiation.^24^

We therefore focused on the molecular basis that underlies the differential regulation. It was previously documented that NKD2 bound to DVL1 and destabilized the latter by enhancing ubiquitin-mediated proteasomal degradation.^27^ Consistently, in our study, NKD2 overexpression accelerated the degradation of DVL1 in undifferentiated cells but not in differentiating cells. We further uncovered that in undifferentiated cells NKD2 up-regulated SFRP1 and SFRP4, while in differentiating cells NKD2 was able to down-regulate SFRP1 and SFRP4.

Both SFRP1 and SFRP4 are secreted factors belonging to the family of SFRPs that antagonize canonical Wnt signaling by competing with frizzled receptors for Wnt ligands.^30^, ^31^, ^32^ SFRP1 and SFRP4 were previously reported to be the regulators of mesenchymal stem/progenitor cell differentiation and bone homeostasis.^33^, ^34^, ^35^, ^36^, ^37^ Therefore, the differential effects of NKD2 on SFRP1 and SFRP4 in undifferentiated versus differentiating cells are probably the molecular basis for the opposite effect of NKD2 on canonical Wnt signaling, although it still needs to be clarified why the different cellular contexts between undifferentiated and differentiating cells lead to such different expression patterns of SFPR1 and SFRP4. Additionally, we further demonstrated that depletion of β-catenin attenuated NKD2-induced deregulation of osteogenic and adipogenic differentiation from mesenchymal progenitor cells. Overall, we conclude that NKD2 regulation of mesenchymal stem/progenitor cells is at least partly attributed to its effect on SFRPs/Wnt/β-catenin signaling.

Of interest, NKD2 overexpression up-regulated osteogenic factors and down-regulated adipogenic factors in undifferentiated progenitor cells, which contradicted the inactive state of canonical Wnt signaling in NKD2-overexpressing undifferentiated cells. This discrepancy suggests that in addition to the Wnt/β-catenin pathway, there exist other mechanisms by which NKD2 regulates differentiation. Bioinformatics analysis revealed the potential interaction between NKD2 and TSC1, which was confirmed by coimmunoprecipitation experiments.

When complexed with TSC2, TSC1 down-regulates mTORC1 signaling.^38^, ^39^, ^40^ Recent emerging evidence has demonstrated that TSC1/mTORC1 signaling plays a role in the differentiation of bone cells and bone homeostasis.^41^, ^42^, ^43^ TSC1 ablation in osterix-expressing osteoblastic cells led to reduced trabecular bone mass, repressed osteogenic differentiation, and enhanced adipogenic differentiation in mice.^41^^,^^42^ In our study, we found that NKD2 interacted with TSC1 and stabilized TSC1 protein level, and inactivated mTORC1 signaling not only in differentiating cells but also in undifferentiated cells. In addition, the depletion of TSC1 attenuated NKD2-induced deregulation of osteogenic and adipogenic differentiation. The data indicate that the role of NKD2 in osteoblast differentiation and adipogenesis also depends on its interaction with TSC1/mTORC1 signaling.

Previous studies have established crosstalk between mTORC1 and Wnt/β-catenin signaling pathways.^44^ Activation of mTORC1 signaling by TSC1 gene deletion down-regulates Wnt/β-catenin signaling in mice.^44^ We also observed that activation of mTORC1 signaling by TSC1 gene depletion inactivated NKD2-regulated Wnt/β-catenin signaling in both undifferentiated and differentiating cells, highlighting an interplay between TSC1/mTORC1 and Wnt/β-catenin signaling pathways that underlies the fine-tuning of mesenchymal cell differentiation by NKD2.

Our next major interest is whether NKD2 gene manipulation in BMSCs has any effect on the balance of osteoblasts and adipocytes and thereby affects bone homeostasis in mice. As expected, the transplantation of NKD2-overexpressing BMSCs to the marrow improved cancellous bone mass in ovariectomized mice. Associated with this was the increase of osteoblasts and reduction of marrow adiposity. Notably, TSC1-positive cells were increased on the trabeculae in OVX mice after the transplantation of NKD2-overexpressing BMSCs, suggesting the involvement of mTORC1 signaling in the *in vivo* role of NKD2.

Of interest, transplantation of NKD2-overexpressing BMSCs led to a reduction of osteoclasts in ovariectomized mice. This suggests that NKD2 in osteoblastic lineage may regulate osteoclast differentiation. In support of this hypothesis, we further demonstrated that NKD2-overexpressing BMSCs suppressed the potential of bone marrow osteoclast precursor cells to differentiate toward osteoclasts, which is associated with the down-regulation of RANKL by NKD2 *in vitro* and *in vivo*. It is known that β-catenin expression in osteoblastic cells is detrimental to osteoclastogenesis through down-regulating the RANKL/osteoprotegerin ratio.^45^ Herein, we also found that depletion of β-catenin in undifferentiated mesenchymal cells increased RANKL expression, an effect opposite to that of NKD2, and compromised the down-regulation of RANKL by NKD2. However, based on the fact that NKD2 inactivated Wnt/β-catenin signaling in undifferentiated cells, we conclude that the regulation of RANKL and osteoclastogenesis by NKD2 in undifferentiated cells is independent of Wnt/β-catenin signaling.

In conclusion, our study has demonstrated that NKD2 plays a role in the cell fate determination of mesenchymal stem/progenitor cells, favoring osteoblast differentiation over adipocyte formation. Mechanistic explorations suggest that the role of NKD2 involves Wnt/β-catenin and mTORC1 signaling pathways. In addition, NKD2 down-regulates RANKL in BMSCs and thereby inhibits osteoclast differentiation. Increasing the level of NKD2 in BMSCs ameliorates cancellous bone loss in ovariectomized mice by increasing osteoblasts and reducing adipocytes and osteoclasts. Taken together, the current research suggests that NKD2 may serve as a novel therapeutic target for metabolic bone disorders such as osteoporosis.

## Author contributions

LS and XL performed the experiments and analyzed the data. XY helped in some experiments. JZ, EZ, and HY helped in designing and supervising the research. XL designed the study and supervised the research. BW designed the study, supervised the research, and wrote the manuscript. All the authors read and approved the final manuscript.

## Data availability

The data that support the findings of this study will be available from the corresponding author upon reasonable request.

## Funding

This work was funded by the 10.13039/501100001809National Natural Science Foundation of China (No. 82072389, 82272444, 81972033).

## Conflict of interests

All authors declared no conflict of interests.
