# Supplementary material for: Naked cuticle homolog 2 controls the differentiation of osteoblasts and osteoclasts and ameliorates bone loss in ovariectomized mice
Source: Genes Dis. 2024 Jan 12;12(1):101209. doi: 10.1016/j.gendis.2024.101209 (PMC11567042; doi:10.1016/j.gendis.2024.101209)
Supplement: Multimedia component 1 [file mmc1.pdf]

Supplemental Figures

Figure S1

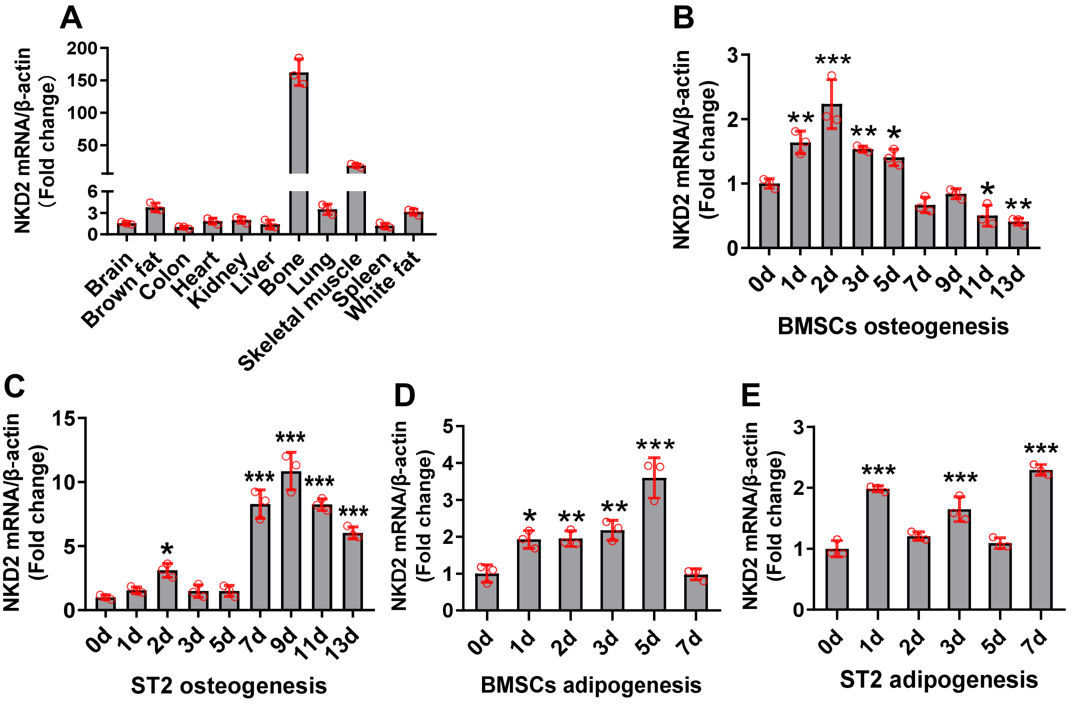

Figure S1. NKD2 was regulated during the differentiation of mesenchymal stem/progenitor cells. The expression level of NKD2 mRNA in various tissues was evaluated by using RT-qPCR (A). The mRNA expression pattern of NKD2 was examined during osteogenic differentiation of primary BMSCs (B) and ST2 cells (C) using RT-qPCR. The mRNA expression pattern of NKD2 was examined during adipogenic differentiation of primary BMSCs (D) and ST2 cells (E) using RT-qPCR. Values represent mean ± SD, n=3. *Significant vs. day 0, *$p<0.05$, **$p<0.01$, ***$p<0.001$.

Figure S2

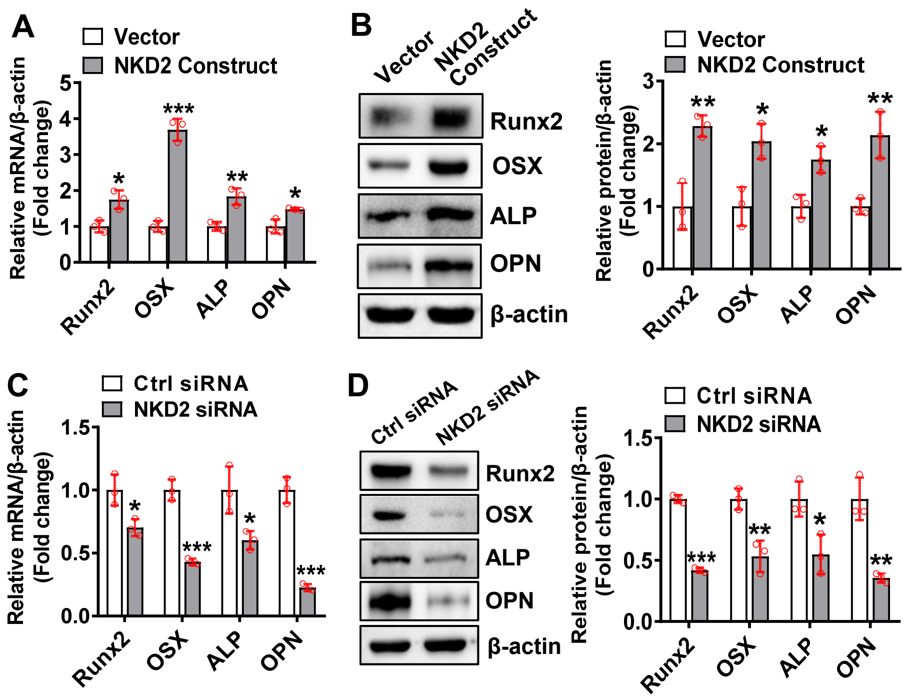

Figure S2. NKD2 upregulated the expression of osteogenic factors. Transfected ST2 cells were grown in osteogenic medium to allow osteoblast differentiation. The mRNA (A, C) and protein (B, D) levels of osteogenic factors were determined at 5 days following osteogenic treatment. Values are mean ± SD, n=3. *p<0.05, **p<0.01, ***p<0.001 vs. vector or control siRNA.

Figure S3

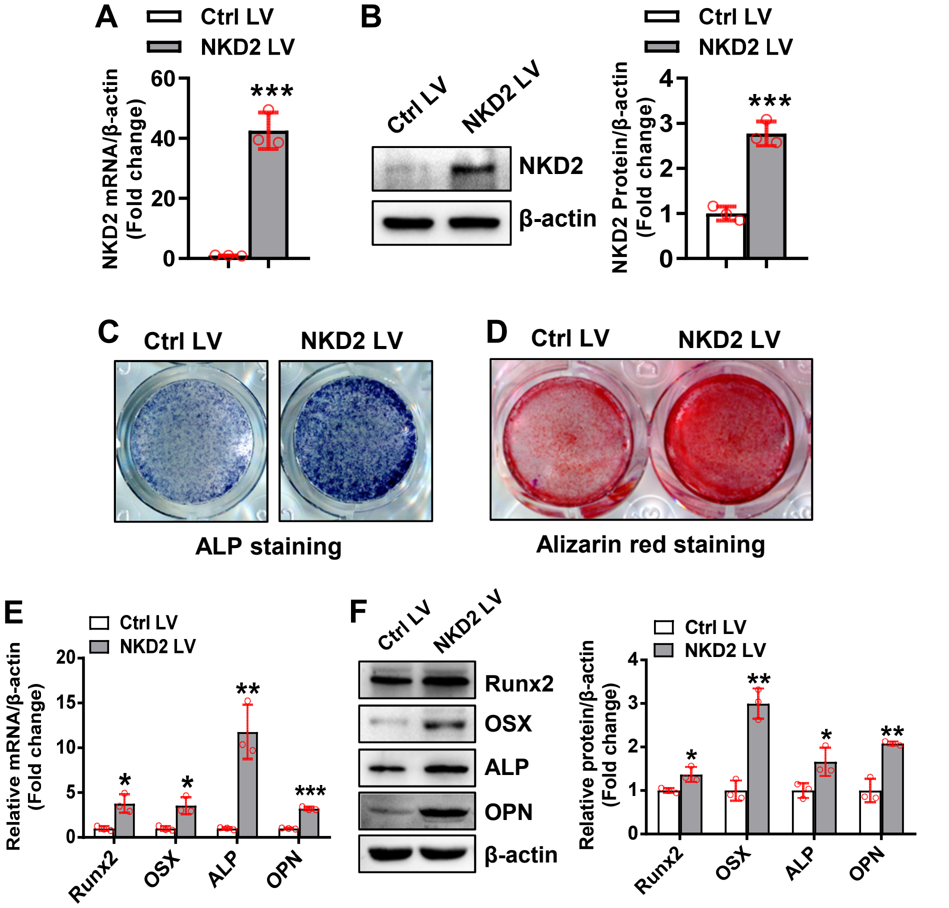

Figure S3. NKD2 promoted osteoblast differentiation from primary BMSCs. Primary BMSCs were infected with NKD2-overexpressing lentivirus (LV). The overexpression of NKD2 was verified using RT-qPCR (A) and Western blotting (B). ALP staining (C) and alizarin red staining (D) were performed on differentiated osteoblasts. The mRNA (E) and protein (F) levels of osteogenic factors were measured 72 h after osteogenic treatment. Values are mean ± SD, n=3. *Significant *vs.* control LV, *p<0.05, **p<0.01, ***p<0.001.

Figure S4

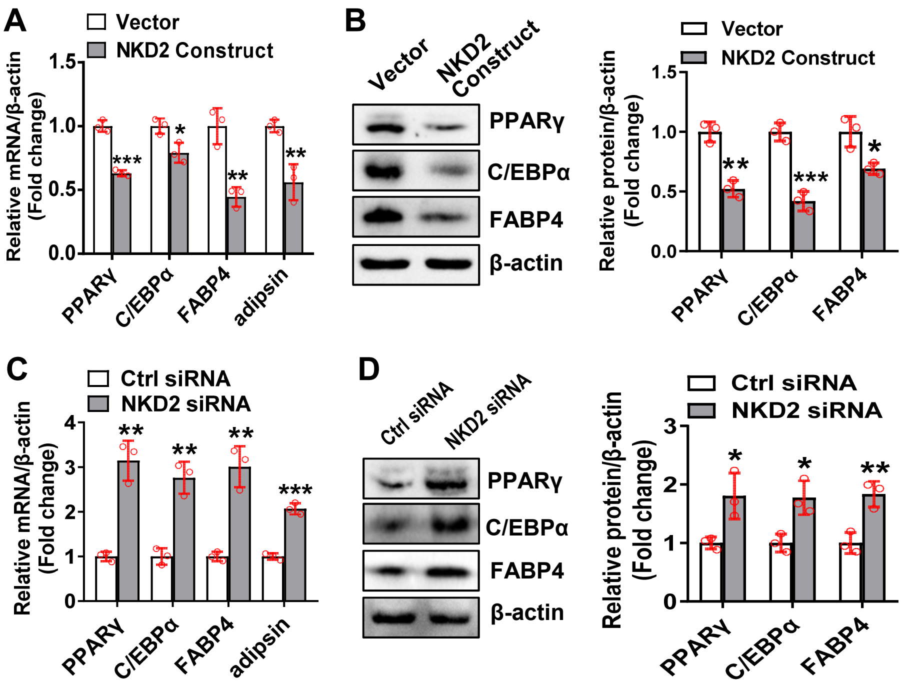

Figure S4. NKD2 downregulated the expression of adipogenic factors. Transfected C3H10T1/2 (A, B) or ST2 (C, D) cells were grown in adipogenic medium to allow adipocyte differentiation. The mRNA (A, C) and protein (B, D) levels of adipogenic factors were determined at 2 days following adipogenic treatment. Values are mean ± SD, n=3. *p<0.05, **p<0.01, ***p<0.001 vs. vector or control siRNA.

Figure S5

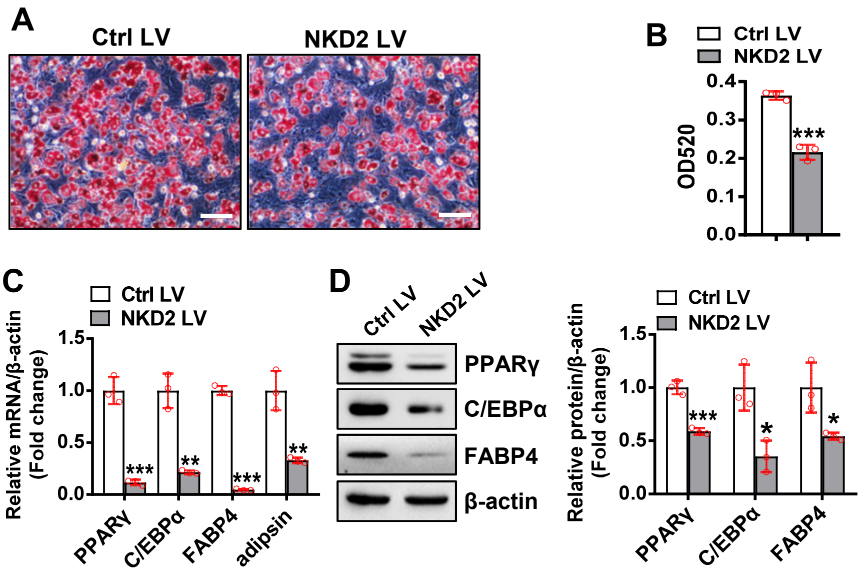

Figure S5. NKD2 suppressed adipocyte differentiation from primary BMSCs. Primary BMSCs were infected with NKD2-overexpressing lentivirus. Oil red O staining was performed on differentiated adipocytes (A). The stain within the cells was extracted with isopropanol, and OD520 was measured (B). The mRNA (C) and protein (D) levels of adipogenic factors were measured 3 days after adipogenic treatment. Image scale in (A): 100 μm. Values are mean ± SD, n=3. *Significant vs. control LV, *$p<0.05$, **$p<0.01$, ***$p<0.001$.

Figure S6

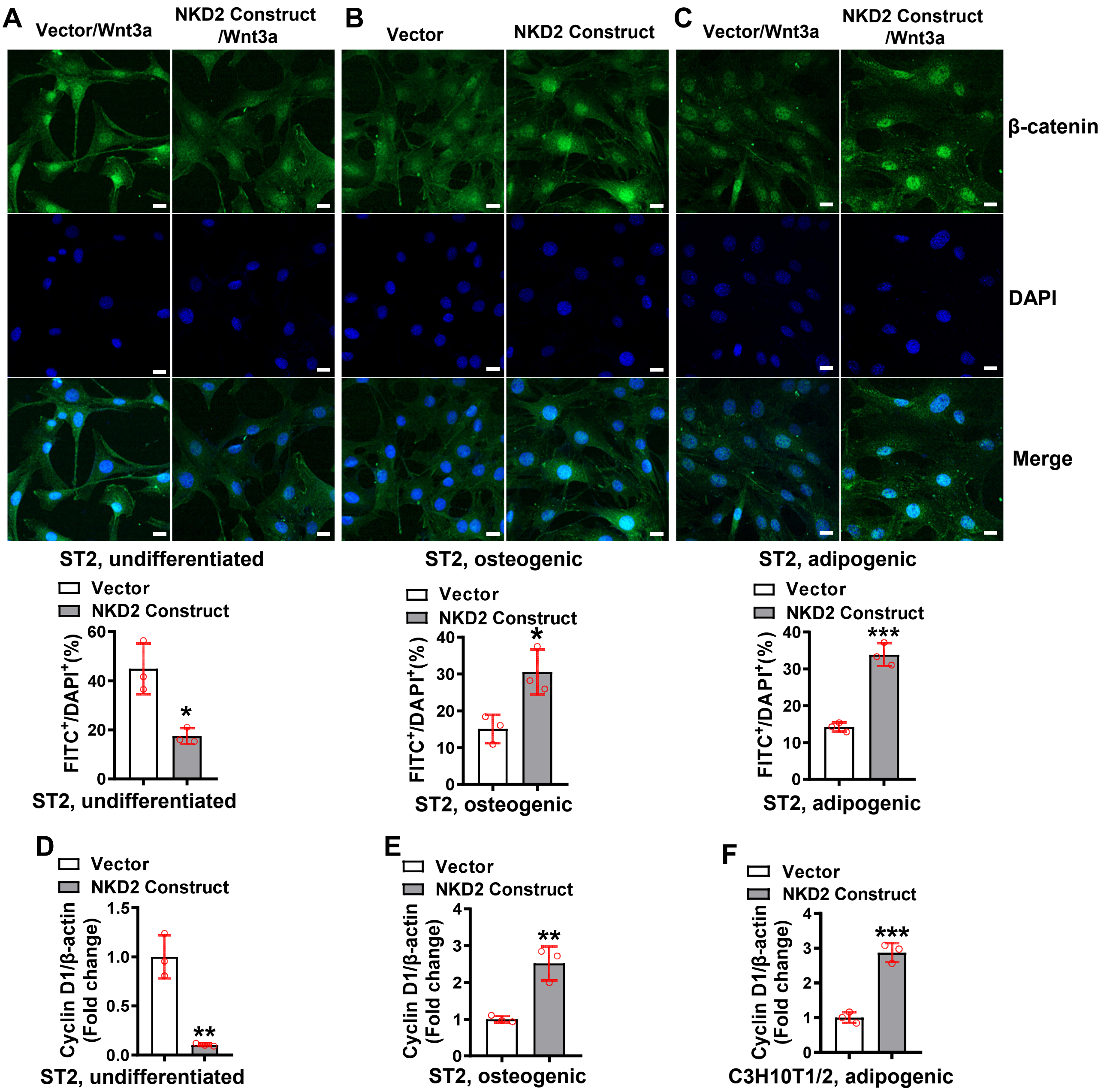

Figure S6. NKD2 differentially regulated the nuclear translocation of β-catenin and cyclin D1 expression in undifferentiated and differentiating cells. ST2 cells were transfected with the NKD2 expression construct or vector for 4 h and then cultured in common medium for 48 h (A), osteogenic medium for 72 h (B), or adipogenic medium for 72 h (C) in the presence or absence of 30 ng/ml Wnt3a as indicated. Immunofluorescence staining was performed to identify the nuclear translocation of β-catenin, and the staining intensity was quantified. Transfected ST2 (D, E) or C3H10T1/2 (F) cells were cultured for 24 h in common medium (D), for 72 h in osteogenic medium (E), or for 72 h in adipogenic medium (F). RT-qPCR was performed to determine the mRNA level of cyclin D1. Scale in (A-C): 20 μm. Values are mean ± SD, n=3. *p<0.05, **p<0.01, ***p<0.001 vs. vector.

Figure S7

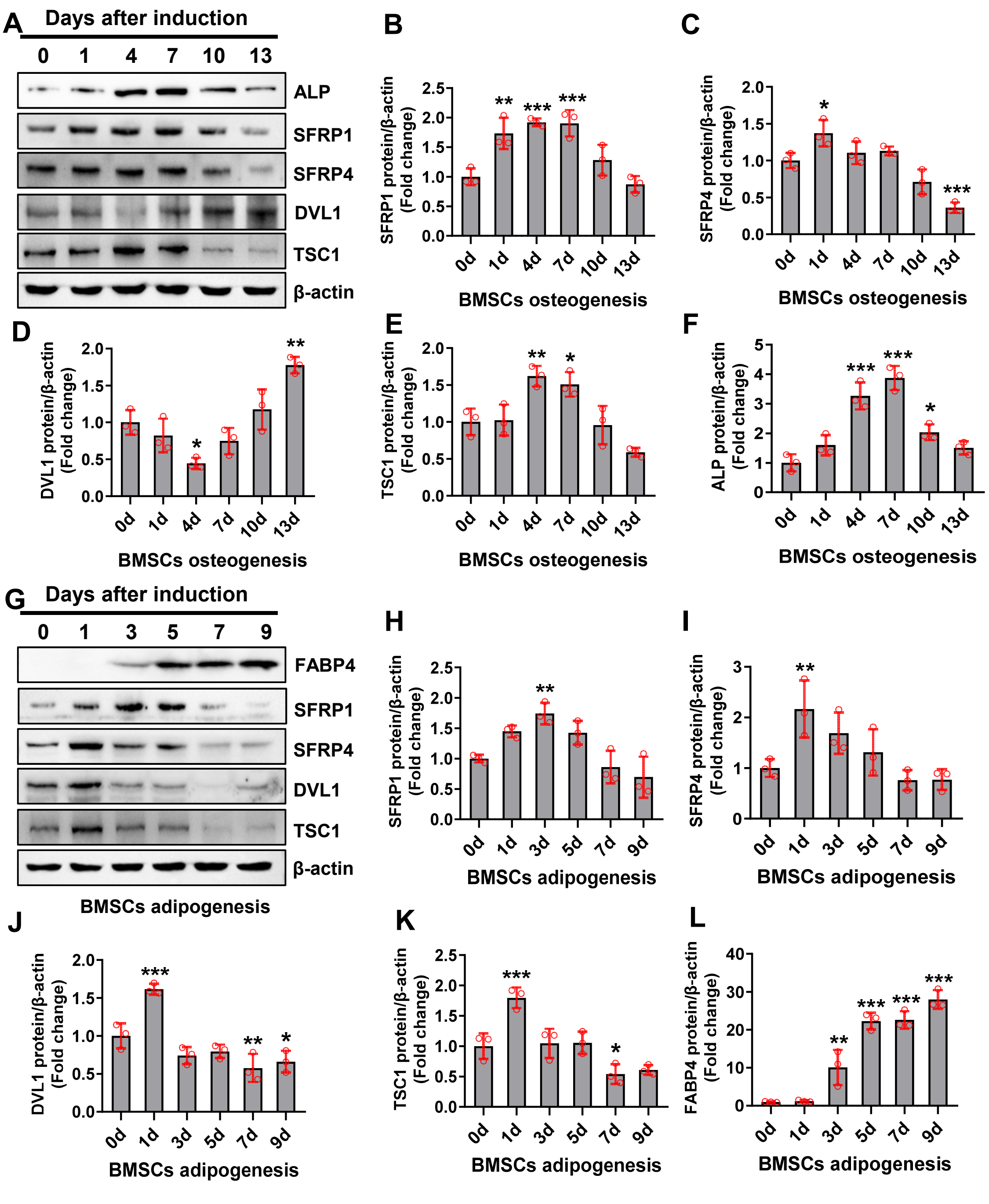

Figure S7. Wnt/β-catenin and mTORC1 signaling proteins were regulated during the differentiation of BMSCs. The protein levels of SFRP1, SFRP4, DVL1 and TSC1 were determined by Western blotting in primary BMSCs during osteogenic differentiation (A-E) or adipogenic differentiation (G-K). The protein levels of ALP (F) and FABP4 (L) were detected as controls. Values represent mean ± SD, n=3. *Significant vs. day 0, *p<0.05, **p<0.01, ***p<0.001.

Figure S8

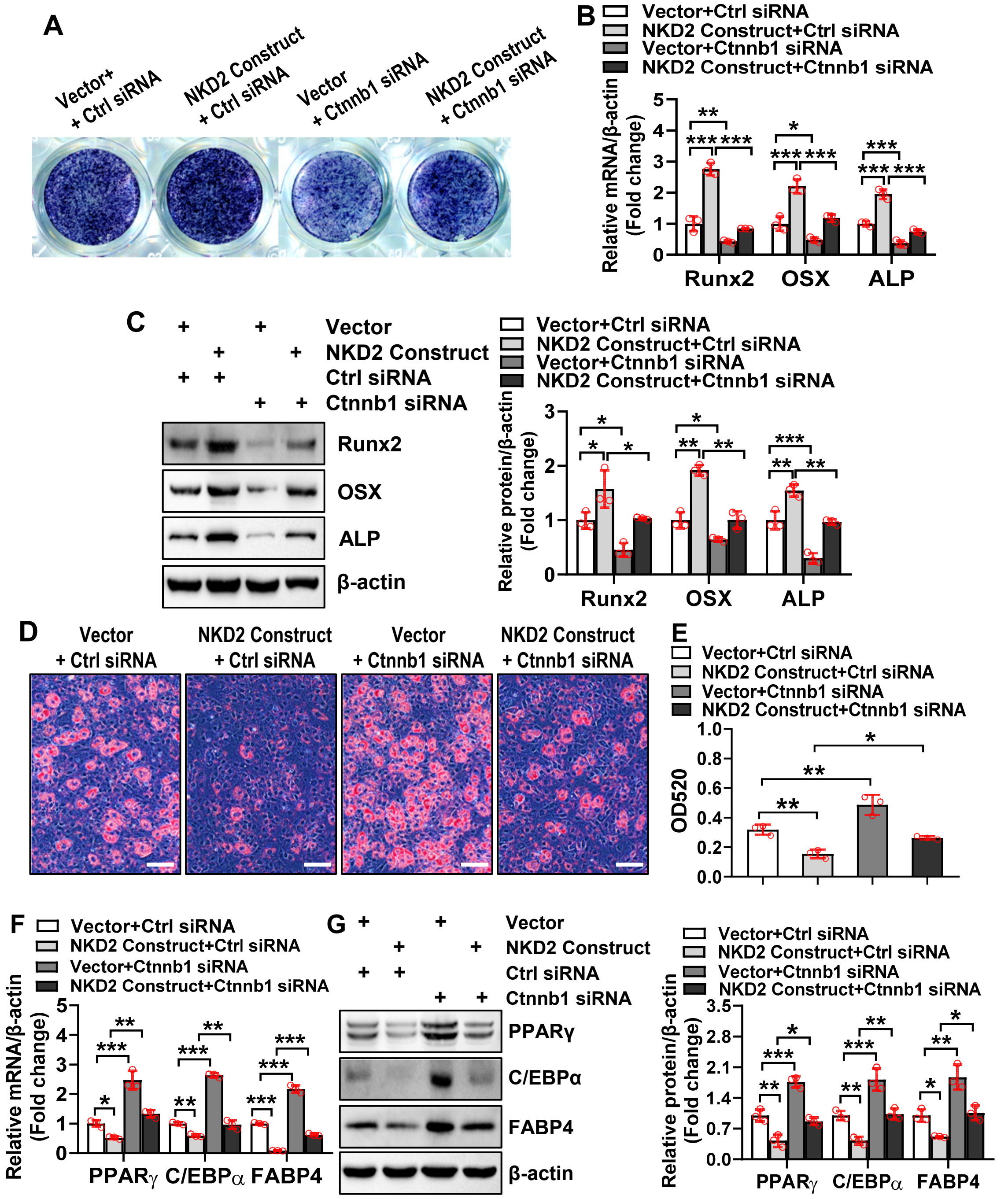

Figure S8. Silencing β-catenin attenuated NKD2-induced deregulation of osteogenic and adipogenic differentiation. ST2 (A-C) and C3H10T1/2 (D-G) cells were cotransfected with the NKD2 expression construct (or vector) and β-catenin siRNA (or control siRNA) and induced to allow osteogenic and adipogenic differentiation, respectively. ALP staining was performed 14 days after osteogenic induction (A). The mRNA (B) and protein (C) levels of osteogenic factors were determined 3 days following osteogenic treatment. Oil red O staining was performed after 5-6 days of adipogenic induction (D). The staining intensity was evaluated by extracting the stain from the cells and measuring OD520 (E). The mRNA (F) and protein (G) levels of adipogenic factors were measured 3 days following adipogenic treatment. Image scale in (D): 100 μm. Values are mean ± SD, n=3. *p<0.05, **p<0.01, ***p<0.001.

Figure S9

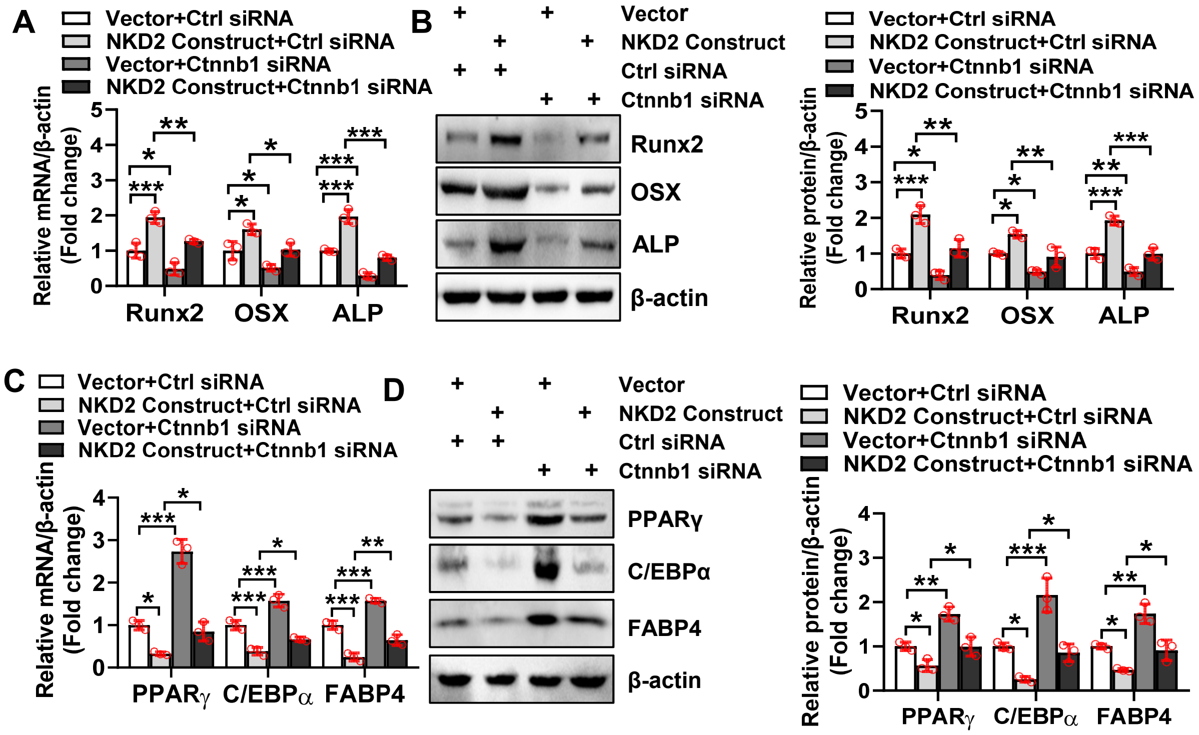

Figure S9. Silencing β-catenin attenuated NKD2-induced deregulation of osteogenic and adipogenic factors. ST2 (A, B) and C3H10T1/2 (C, D) cells were cotransfected with the NKD2 expression construct (or vector) and β-catenin siRNA (or control siRNA) and induced to allow osteogenic or adipogenic differentiation, respectively. The mRNA (A) and protein (B) levels of osteogenic factors were determined 5 days following osteogenic induction. The mRNA (C) and protein (D) levels of adipogenic factors were determined 2 days following adipogenic induction. Values are mean ± SD, n=3. *p<0.05, **p<0.01, ***p<0.001.

Figure S10

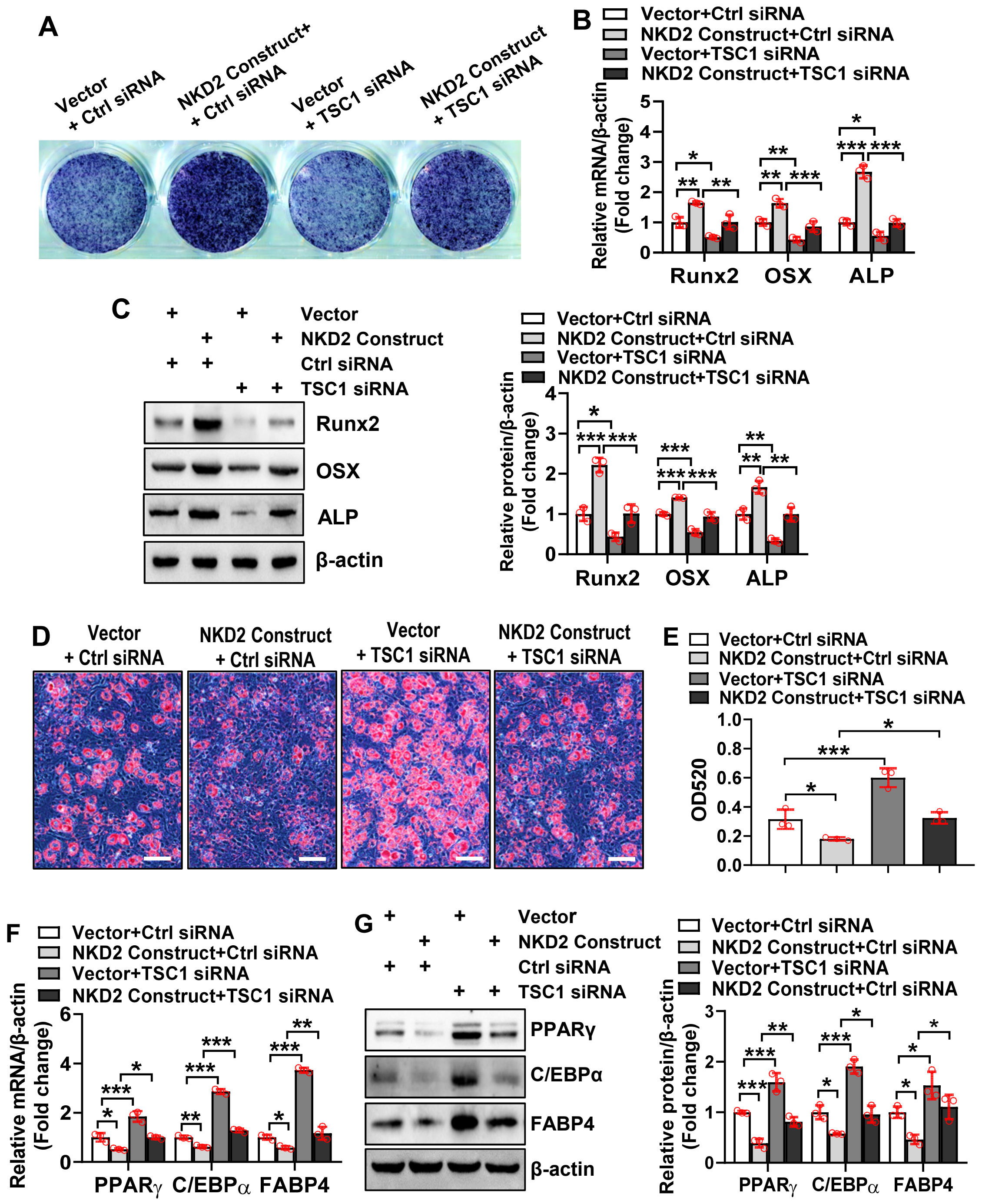

Figure S10. Silencing TSC1 attenuated NKD2-induced deregulation of osteogenic and adipogenic differentiation. ST2 (A-C) and C3H10T1/2 (D-G) cells were cotransfected with the NKD2 expression construct (or vector) and TSC1 siRNA (or control siRNA) and induced to allow osteogenic (A-C) or adipogenic (D-G) differentiation, respectively. ALP staining was performed 14 days after osteogenic induction (A). The mRNA (B) and protein (C) levels of osteogenic factors were determined 3 days following osteogenic treatment. Oil red O staining was performed after 5-6 days of adipogenic induction (D). The staining intensity was evaluated by extracting the stain from the cells and measuring OD520 (E). The mRNA (F) and protein (G) levels of adipogenic factors were determined 3 days following adipogenic treatment. Image scale in (D): 100 μm. Values are mean ± SD, n=3. *p<0.05, **p<0.01, ***p<0.001.

Figure S11

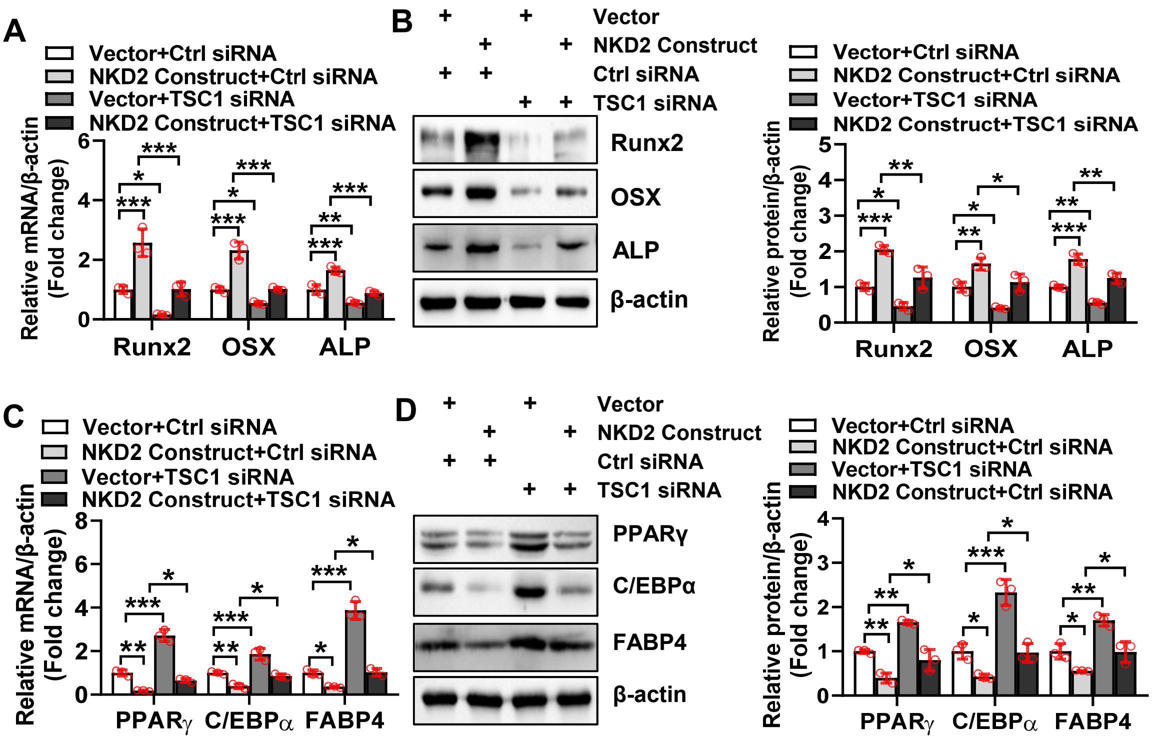

Figure S11. Silencing TSC1 attenuated NKD2-induced deregulation of osteogenic and adipogenic factors. ST2 (A, B) and C3H10T1/2 (C, D) cells were cotransfected with the NKD2 expression construct (or vector) and TSC1 siRNA (or control siRNA) and induced to allow osteogenic or adipogenic differentiation, respectively. The mRNA (A) and protein (B) levels of osteogenic factors were determined 5 days following osteogenic induction. The mRNA (C) and protein (D) levels of adipogenic factors were determined 2 days following adipogenic induction. Values are mean ± SD, n=3. *p<0.05, **p<0.01, ***p<0.001.

Figure S12

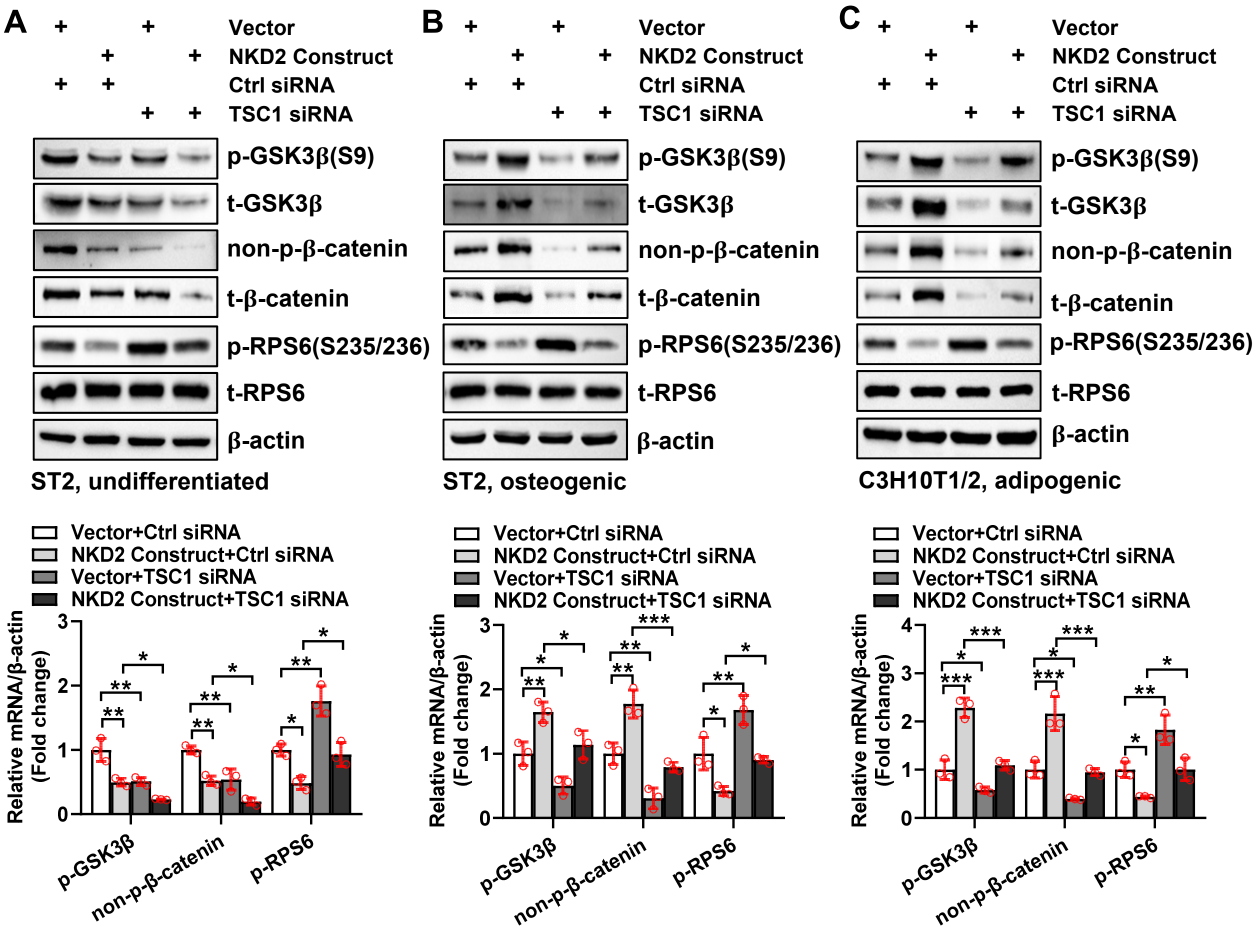

Figure S12. Depletion of TSC1 inactivated NKD2-regulated Wnt/β-catenin signaling in undifferentiated and differentiating cells. ST2 (A, B) and C3H10T1/2 (C) cells were cotransfected with the NKD2 expression construct (or vector) and TSC1 siRNA (or control siRNA) and induced to allow osteogenic or adipogenic differentiation, respectively. Western blotting was performed to measure the protein levels of GSK3β, β-catenin and RPS6. Values are mean ± SD, n=3. *$p<0.05$, **$p<0.01$, ***$p<0.001$.

Figure S13

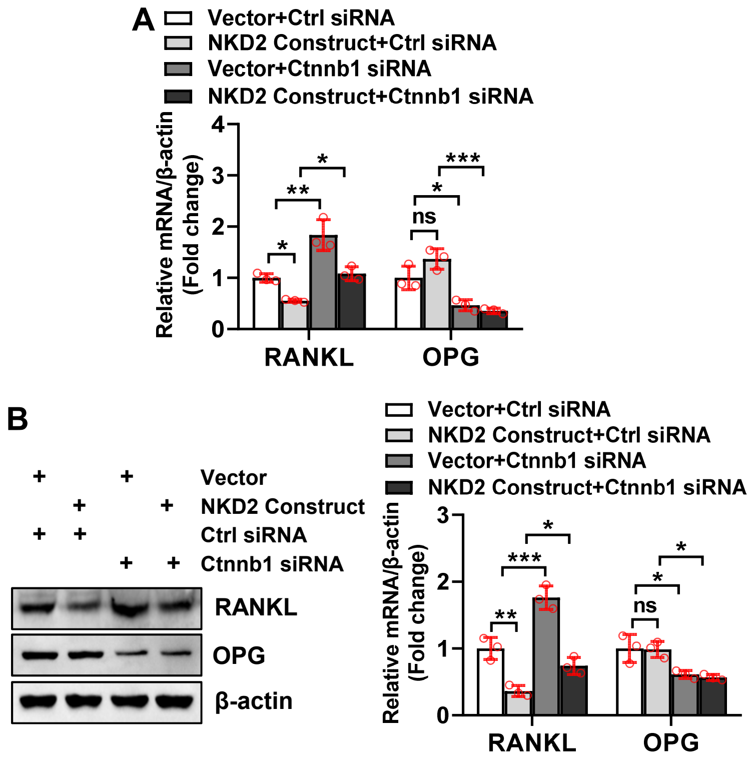

Figure S13. Silencing β-catenin compromised the downregulation of RANKL by NKD2. ST2 cells were cotransfected with the NKD2 expression construct (or vector) and β-catenin siRNA (or control siRNA). The mRNA (A) and protein (B) levels of RANKL and OPG were determined 48 h following transfection. Values are mean ± SD, n=3. *$p<0.05$, **$p<0.01$, ***$p<0.001$.

Supplemental tables

Table S1. siRNA sequences used for gene silencing

Gene	Sense sequence	Antisense sequence
NKD2 siRNA	GCAAGAAGCACUUGAACAUTT	AUGUUCAAGUGCUUCUUGCTT
TSC1 siRNA	GCAGAUGGAGUUCUUGAAUTT	AUUCAAGAACUCCAUCUGCTT
β-catenin siRNA	GGACCUACACUUAUGAGAATT	UUCUCAUAAGUGUAGGUCCTT

Table S2. Primers used for qRT-PCR and cloning

Genes	Forward primer sequences:	Reverse primer sequences
Runx2	TCCTGTAGATCCGAGCACCA	CTGCTGCTGTTGTTGCTGTT
ALP	GTTGGGGGTGCCCACGGT	CCTTGGACAGAGCCATGTATG
Osterix	GGCTTTTCTGCGGCAAGAGGTT	CGCTGATGTTTGCTCAAGTGGTC
OPN	ACTCCAATCGTCCCTACAGTC	GACTCACCGCTCTTCATGTG
PPARγ	CTTGACAGGAAAGACAACGG	GCTTCTACGGATCGAAACTG
C/EBPα	CTGATTCTTGCCAAACTGAG	GAGGAAGCTAAGACCCACTAC
FABP4	AAATCACCGCAGACGACAGG	GGCTCATGCCCTTTCATAAAC
adipsin	ATCCTGGAGCGGCTGTATG	ATCCGTCACTCCATCCATGC
β-actin	TCAGGTTACTGGTTCGGTCTG	ACCAGAGGCATACAGGGACAG
NKD2	CTCCAGCAAGAAGGAATGTCCTC	GTAGGCGGATAGCCTTCTGTCA
NKD2 primers for pcDNA3.1 cloning	TTGGTACCGAGCTCGGATCCGCCAC CATGGGGAAATTTCAGTCCAA	GCTGGATATCTGCAGAATTCCTA GGATGGGTGGAAGTGGTGGT
NKD2 primers for lentiviral clonng	ATTCTAGAGCTAGCGAATTCGCCAC CATGGGGAAATTTCAGTC	AGATCCTTGCGGCCGCGGATCCC TAGGATGGGTGGAAGTGGT

Table S3. Antibodies Used for Western blotting

Antibody	Catalog number	Brand
anti-S6K1	ab32529	Abcam(Cambridge, MA, USA)
anti-LRP6	ab134146	
anti-SFRP4	A6409	Abclonal (Wuhan, China)
anti-mTOR	A11354	
anti-RPS6	A6058	
anti-β-actin	AC026	
anti-TSC1	A0720	
anti-NFATc1	A1539	
anti-cathepsin K	A1782	
anti-FABP4	67167-1-Ig	Proteintech (Wuhan, China)
anti-osteopontin	22952-1-AP	
anti-DVL1	27384-1-AP	
anti-GSK3β	24198-1-AP	
anti-phospho-GSK3β	67558-1-Ig	
anti-SFRP1	26460-1-AP	
anti-phospho-mTOR	67778-1-Ig	
anti-4EBP1	60246-1-Ig	
anti-β-catenin	66379-1-Ig	
anti-phospho-S6K1	9234S	Cell Signaling Technology (Danvers, MA, USA)
anti-phospho-RPS6	#2211	
anti-phospho-4EBP1	#9451	
anti-non-phospho-β-catenin	#8814	
anti-C/EBPα	#8178	
anti-Runx2	#12556	
anti-PPARγ	#2443	
anti-phospho-LRP6 (Ser1490)	#2568	
anti-NKD2	AF4647	Affinity (Changzhou, China)
anti-osterix	DF7713	
anti-TCF7L2	DF7622	
anti-ALP	DF6225	
anti-RANKL	HA500369	Huabio (Hangzhou, China)
anti-osteoprotegerin	BS6684	Bioworld (Bloomington, MN, USA)
